# Controversies and expectations for the prevention of GVHD: A biological and clinical perspective

**DOI:** 10.3389/fimmu.2022.1057694

**Published:** 2022-11-23

**Authors:** Benjamin Watkins, Kirsten M. Williams

**Affiliations:** Aflac Cancer and Blood Disorders Center, Children’s Healthcare of Atlanta, Emory University, Atlanta, GA, United States

**Keywords:** GVHD, GVHD prophylaxis, controversy, GVHD biology, GVHD therapies

## Abstract

Severe acute and chronic graft versus host disease (GVHD) remains a major cause of morbidity and mortality after allogeneic hematopoietic cell transplantation. Historically, cord blood and matched sibling transplantation has been associated with the lowest rates of GVHD. Newer methods have modified the lymphocyte components to minimize alloimmunity, including: anti-thymocyte globulin, post-transplant cyclophosphamide, alpha/beta T cell depletion, and abatacept. These agents have shown promise in reducing severe GVHD, however, can be associated with increased risks of relapse, graft failure, infections, and delayed immune reconstitution. Nonetheless, these GVHD prophylaxis strategies have permitted expansion of donor sources, especially critical for those of non-Caucasian decent who previously lacked transplant options. This review will focus on the biologic mechanisms driving GVHD, the method by which each agent impacts these activated pathways, and the clinical consequences of these modern prophylaxis approaches. In addition, emerging novel targeted strategies will be described. These GVHD prophylaxis approaches have revolutionized our ability to increase access to transplant and have provided important insights into the biology of GVHD and immune reconstitution.

## Current understanding of the mechanism underlying acute GVHD

**Figure 1:**
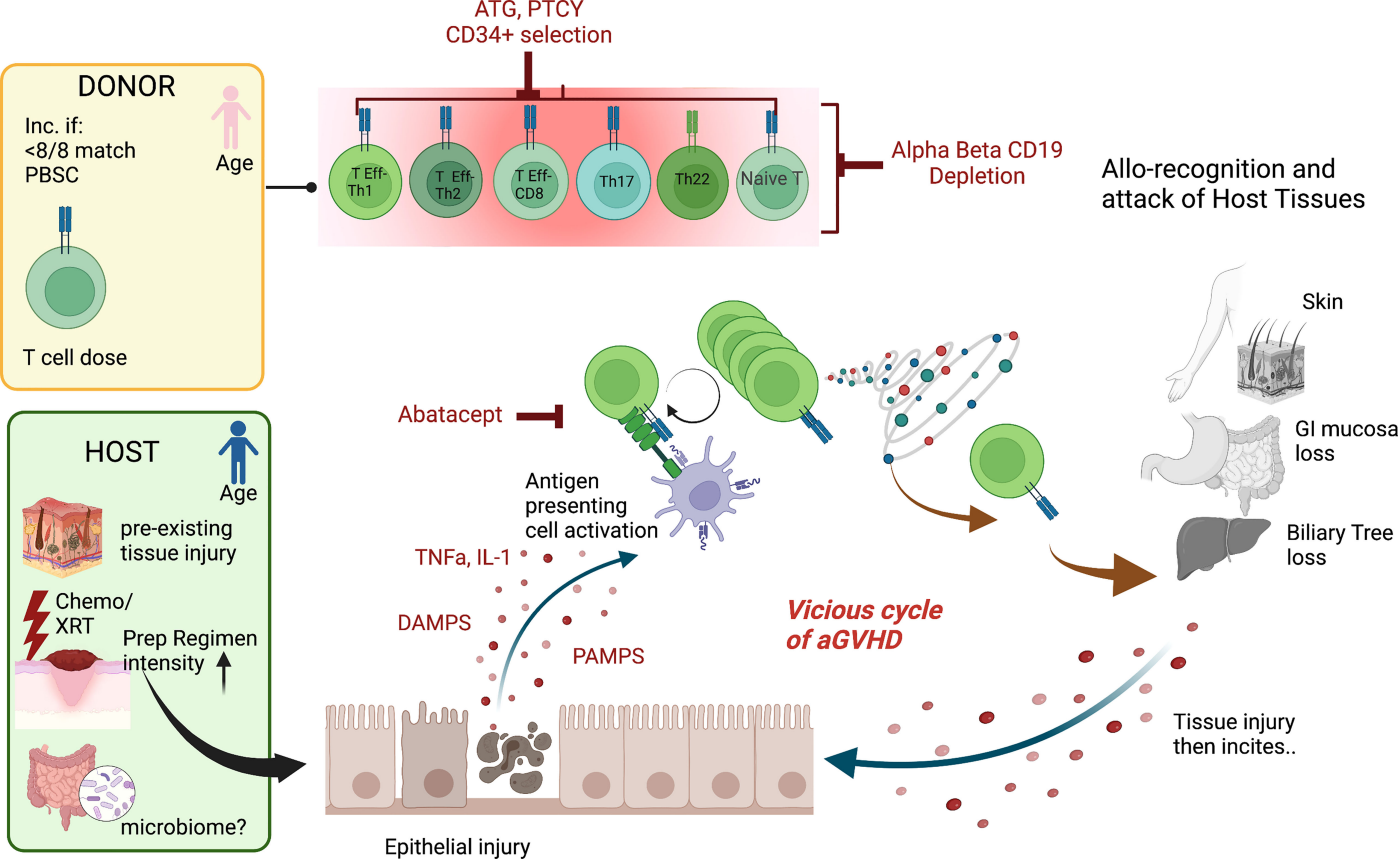
Mechanisms of aGVHD Schema of the contributions to acute GVHD after hematopoietic cell transplantation The pink donor and blue recipient represent the gender mismatch (female to male); <8/8 match= not fully HLA matched donor/recipient pair, PBSC, peripheral blood stem cell source; T cell dose, higher T cell doses are linked to more disease. For the host, chemo/XRT, chemotherapy/radiation with increased preparative regimen intensity linked to increased risk of aGVHD and the microbiome potentially playing a role. The key contributing T cell populations are shown (T Eff-Th, T effector helper cell; DAMPS, danger-associated molecular patterns; PAMPS, pathogen-associated molecular patterns. The figures were generated by the authors using BioRender.com.

While significant advances have been made in our knowledge of the pathogenesis of acute graft versus host disease (aGVHD), our understanding is incomplete. Acute GVHD is thought to primarily be caused by the recognition of self (or host) antigens by donor T cells. Tissue damage is a key component to initiate aGVHD and often begins prior to the introduction of the graft. This tissue injury is due to inflammation associated with the HCT preparative regimen but may also have occurred as part the underlying disease (e.g., hemophagocytic lymphohistiocytosis) or its treatment (e.g., chemotherapy for hematologic malignancy). Myeloablative and total body irradiation-based regimens associated with greater tissue inflammation have been linked to higher rates of aGVHD (1, 2). Notably, the sites most affected by aGVHD are also the sites of greatest tissue injury after HCT, the skin, gastrointestinal tract, and liver. This tissue damage leads to the activation of antigen presenting cells (APCs) through the release of inflammatory cytokines (TNF, IL-1, and others), pathogen-associated molecular patterns (PAMPs), danger-associated molecular patterns (DAMPs), and the increased expression of MHC antigens and costimulatory pathways (3–6). This initiates donor T cell activation that target host antigens *via* host or donor antigen presentation (via MHC) to donor T cells, followed by T cell receptor (TCR) engagement and costimulation in the established proinflammatory milieu (7). This process is augmented in the setting of antigen mismatch and increased T cell dose but can occur even in matched transplants due to the absence of host thymus selection by donor incoming T cells and recognition of minor antigen differences (8, 9). These activated T cells will undergo proliferation and differentiation and produce more pro-inflammatory cytokines, PAMPs, and DAMPs, leading to an inflammatory cascade that results in the end-organ damage known as aGVHD (3). aGVHD has traditionally been thought to be a donor CD4+ T cell mediated disease, specifically Th1 and Th17 driven. However, donor CD8+ T cells alone can be sufficient to induce aGVHD (10) and Th2 and Th22 cells may play an important role as well (11, 12). Additionally, host tissue-resident T cells have also been implicated in the development of aGVHD (13). Murine studies have suggested that naïve T cells are the primary mediators of aGVHD (14–16), though this has yet to be confirmed clinically. Many risk factors for aGVHD have been identified and include increased HLA disparity, increased intensity of the conditioning regimen, female donors for male recipients, and decreased gut bacterial diversity (17–22). In summary, aGVHD likely stems from tissue injury, inciting a proinflammatory milieu with host antigen presentation, T cell activation that includes many CD4+ subsets, and a vicious cycle of T cell produced proinflammatory cytokines that induce ongoing tissue damage (**Figure 1****)**.

## Current understanding of the mechanism underlying chronic GVHD

**Figure 2:**
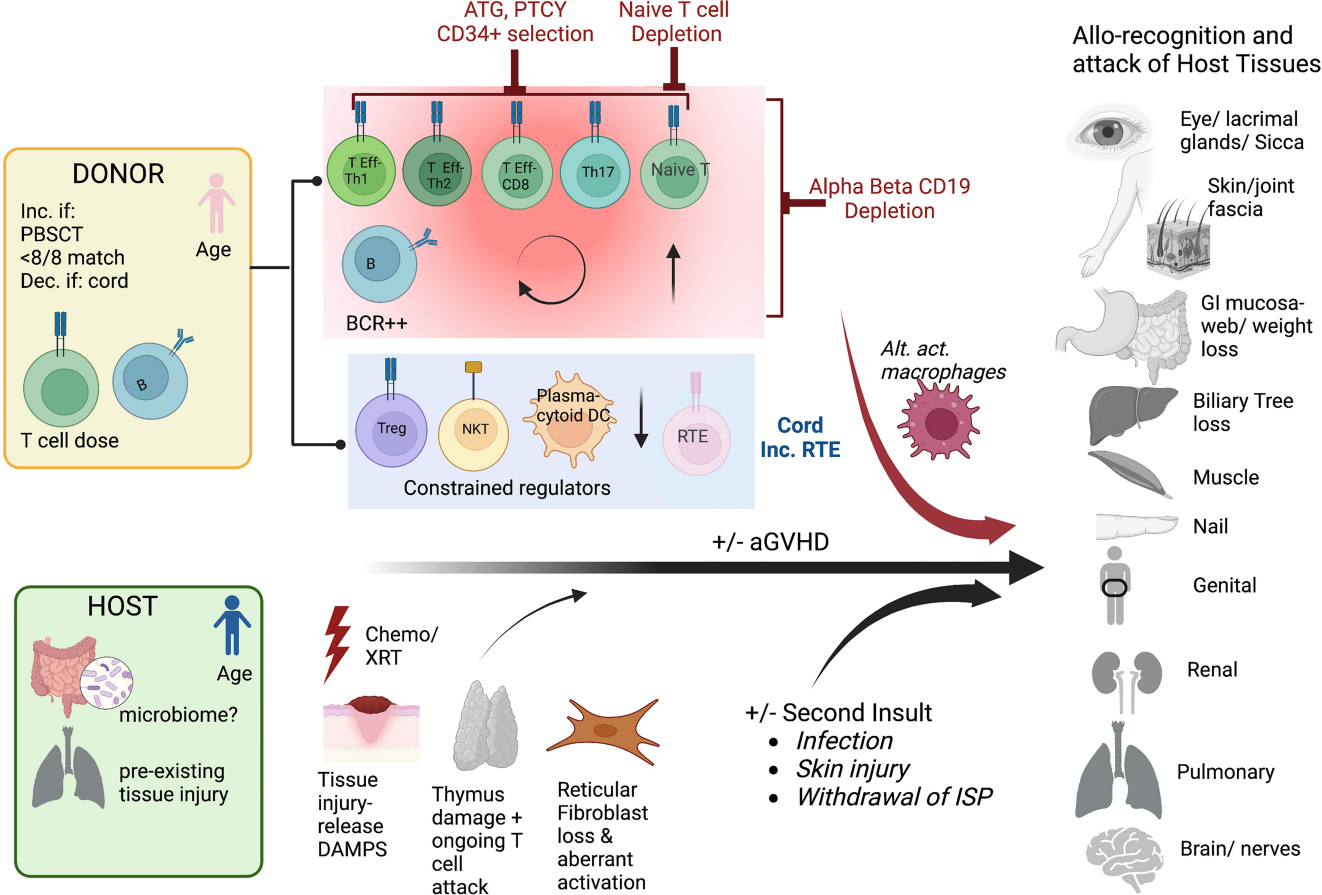
Mechanisms of cGVHD Schema of the contributions to chronic GVHD after hematopoietic cell transplantation The pink donor and blue recipient represent the gender mismatch (female to male); <8/8 match= not fully HLA matched donor/recipient pair, PBSCT= peripheral blood stem cell source, dec. if cord= decreased if cord blood source used, T cell dose= higher T cell doses and B cells are linked to more cGVHD. For the host, the microbiome may potentially play a role in addition to pre-HCT organ injury. The key contributing T cell populations are shown (T Eff-Th, T effector helper cell), Treg, T regulatory cell; RTE, recent thymic emigrants; DAMPS, danger-associated molecular patterns; alt. act. Macrophages, alternatively activated macrophages. The figures were generated by the authors using BioRender.com.

The mechanisms underlying chronic GVHD remain incompletely understood. Since acute GVHD is a major risk factor for the development of cGVHD, there is overlap in these drivers. These include tissue injury, T cell dose, and age of the donor and recipient, and withdrawal of immunosuppression (**Figure 2****)**. Tissue injury may be pre-existing, due to pre-transplant preparative regimens such as in aGVHD, or due to second insults, induced by later infections and the impact on the microbiome, or local injury e.g. sun-exposure (23). Similar to aGVHD, tissue injury leads to the production of DAMPS, exposing previously dormant host antigens that can engender alloimmunity (24). Higher T cell dose, HLA mismatch, and use of peripheral blood mobilized stem cells rather than marrow are risk factors for both acute and chronic GVHD (17, 25, 26). This is likely due to presence of higher numbers of alloreactive T cells in the product, and a greater likelihood of alloreactivity due to mismatch. Increasing age of the donor and recipient also increase the likelihood of acute and chronic GVHD, which may be due to greater exposure to host antigens through increased age-related tissue impairment and a more oligoclonal donor T cell populations, linked to decreased tolerance. Advancing age is also linked to impaired thymopoiesis post-HCT, which may explain part of the increased risk of cGVHD, as T cell recovery is then reliant upon peripheral expansion rather than recovery of thymus activity leading to increased diversity and tolerant T cells (27–30). However, strategies to reduce aGVHD have not always decreased cGVHD, and vice versa, suggesting that there are unique biologic pathways that can incite cGVHD. Cord blood T cells with a predominance of recent thymic emigrants, and male donors for male recipients have both been associated with less cGVHD, likely due to increased tolerance of the infused lymphoid populations (31–34). Dysregulated T subpopulations have been specifically linked to cGVHD including a preponderance of Teff and Th17s, or low Treg/Teff and NKT/Teff ratios (35–39). Miscreant B cells and a paucity of plasmacytic dendritic cells have similarly been linked to cGVHD propagation (40–46). Alternatively activated macrophages have also been linked to some cGVHD processes (47). Finally, the role of host stromal cells in damaged secondary lymphoid organs may set up pathologic immune responses driving cGVHD, through aberrant interactions with CD4 T cells and B cells (40, 48–50). Collectively, these data support that cGVHD is a dysregulated immunity, often established early in the transplant process, through tissue destruction, alloantigen recognition, and a predominance of activating lymphocytes with few regulator cells to curb the maelstrom that culminates in chronic GVHD.

## Strategies to reduce acute and/or chronic GVHD

One of the most important factors in the prevention of GVHD is the selection of a donor. HLA identical matched siblings remains the gold standard for transplantation and is associated with the lowest rates of acute and chronic GVHD. There is ongoing debate over the next best option (unrelated, cord blood, or haploidentical donor) which will be discussed. High resolution HLA typing for unrelated donors is recommended to include HLA-A, HLA-B, HLA-C, HLA-DRB1, and HLA-DPB1 loci with HLA-DQB1 and DRB3/4/5 optional (51). Some studies have shown that mismatches at HLA-DQB1 (52), HDRB3/4/5 (53), and others may increase AGVHD however this has not been validated in larger studies and has not been shown to impact survival. Matching at HLA-A, HLA-B, HLA-C, and HLA-DRB1 should be prioritized and nonpermissive HLA-DPB1 mismatches should be avoided due to its association with increased mortality (52). HLA mismatching at HLA-A, HLA-B, HLA-C, and HLA-DRB1 is associated with increased rates of GVHD and decreased survival but there is no agreed open preference for mismatched loci or allele combination other than prioritizing HLA-C*03:03 over C*03:04 (51). More recently evidence has also suggested that certain HLA-B leader dimorphisms may impact outcomes, with better rates of GVHD and nonrelapse mortality for those mismatched at the threonine leader (TT genotype) compared to either the methionine genotypes (MM or MT) (54).

**Figure 3:**
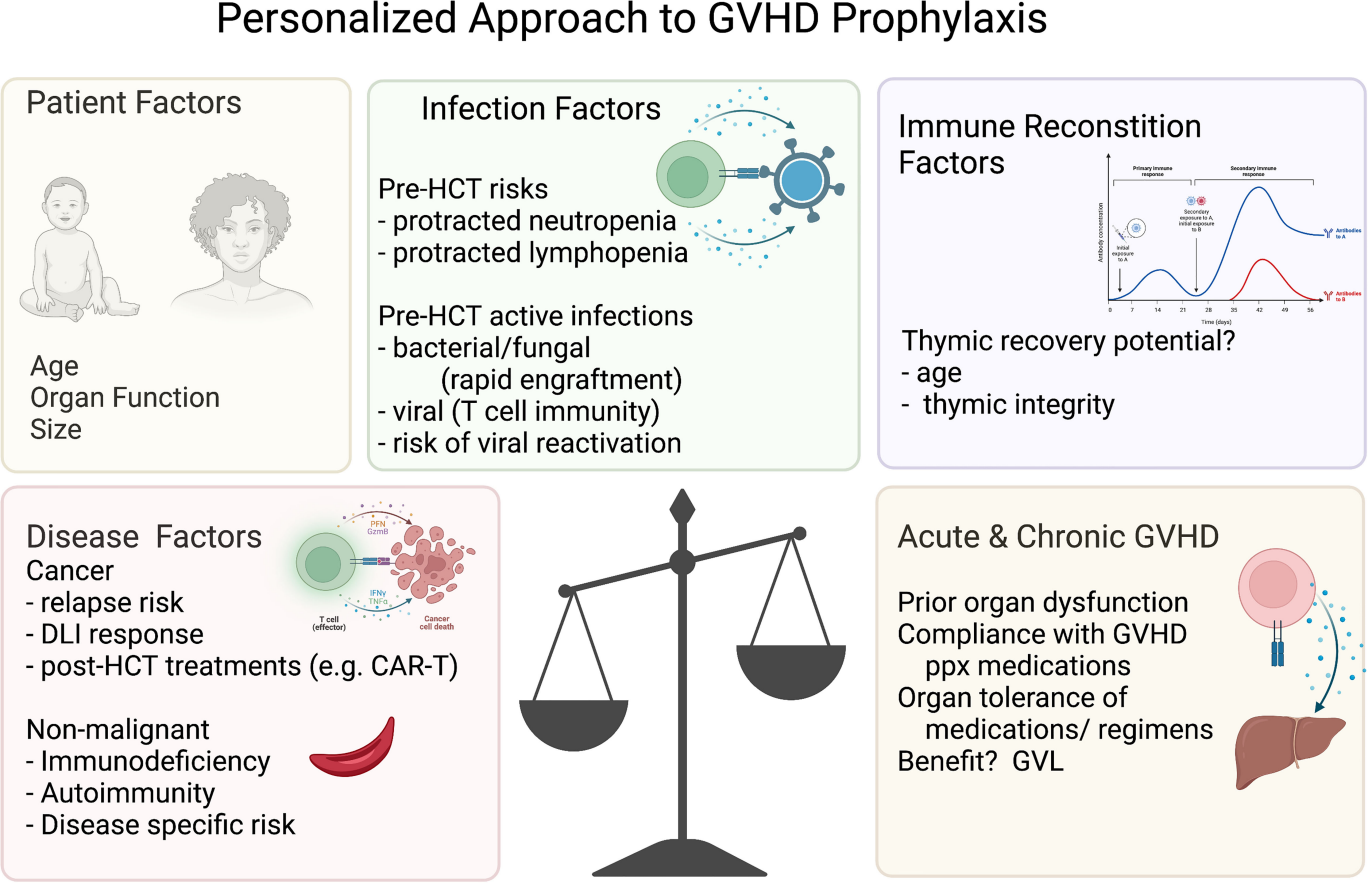
Personalized approach to GVHD prophylaxis. Variables to consider for the personalized approach to GVHD prophylaxis. The figures were generated by the authors using BioRender.com.

Other strategies to reduce acute and/or chronic GVHD have focused on preparative regimens and graft manipulation. In addition to the use of immature thymus-derived T cells in cord blood, these have largely included either prioritizing genetically HLA-matched donors and/or graft engineering *via ex vivo* and *in vivo* lymphocyte selection, depleting functional malevolent lymphocytes to achieve long term durable tolerance. The potential risks of this approach that curtails the infused lymphocyte activity are increased relapse rates, graft failure, or infections (**Figure 3****)**. The application of the GVHD-free, relapse free, survival proportion has enhanced our ability to compare these GVHD-reduction approaches, including these key risks in a single outcome measure, and thus included in the discussions below where possible. However, it is important to note, that the risks of GVHD and relapse may be different in terms of long-term outcomes, so there remains value in reporting rates of relapse and acute and chronic GVHD separately. Herein, we describe the published data for each method of GVHD reduction and then discuss the controversies in the field between the disparate donor and prophylaxis regimen options.

## Anti-thymocyte globulin

Anti-thymocyte globulin (ATG) is composed of antibodies to T cells generated in a horse or rabbit and thus work by antibody mediated clearance of T cells. ATG is typically added to a standard backbone of methotrexate and calcineurin inhibitor though rare studies include other agents (e.g., sirolimus). The equine preparation was generated against human thymus cells. Rabbit ATG is generated by inoculating rabbits with either a human T cell leukemia cell line (ATG-F) or human thymocytes (ATG-T). Because these antibodies are developed in response to different T cell populations, it is not surprising that the polyclonal antibody composition differs. While ATG-F and ATG-T both react to CD2, CD8, CD11a, CD107a, ATG-F has a higher response to CD28 and ATG-T has greater activity against CD3 and CD4 (55). Data have suggested that rabbit ATG may preserve Tregs better than equine ATG, a potential benefit to avert GVHD (56, 57). While equine ATG is still utilized in some preparative regimens, most current practice utilizes rabbit ATG as some larger studies have shown that the rabbit preparation was associated with less acute and chronic GVHD, likely due to better T cell depletion (58, 59). While the effect of rabbit ATG on T cell subpopulations is not fully understood in humans *in vivo*, one study linked reduced cGVHD to lower naïve helper T cells after ATG exposure, and suggested that this was a primary mechanism of action (60). The impact of ATG on immunity is also influenced by the total dose, timing of administration in relation to transplantation, and peri-transplant host lymphocyte count. Higher doses of ATG, timing close to transplantation, and lower host total lymphocyte count can all lead to persistent ATG exposure after the infusion of donor T cells and subsequently to the downstream effects of donor T cell depletion, increasing the potential for relapse, infections, and post-transplant lymphoproliferative disorders (61–66). Thus, these factors must be considered when evaluating outcomes after ATG.

Studies have consistently shown reduction of acute and chronic GVHD after ATG exposure. Most studies are performed using peripheral blood, many randomized to controls, and most include matched related and unrelated donors (67–76). These studies represent over 1600 adult patients with hematologic malignancies treated with ATG, with the majority receiving ATG-F, with few receiving ATG-T. The median rates of grade II-IV aGVHD were 18% across the studies (ranging from 7-34%), and 28% cGVHD (16-28%) with 12% extensive cGVHD (6-13%), lower than controls in randomized studies (67–76). The relapse rates were not significantly increased in most studies, ranging from 16-37%, with higher rates of relapse often linked to higher exposure of ATG (67–76). In most studies, infections were comparable between ATG treated patients and other GVHD prophylaxis strategies, with severe bacterial infections in 42-45% of patients reported (2 studies), invasive fungal disease in 14% (2 studies), and deaths to due to infections ranging from 15-17% of the total (67–76). Notably, the incidence of viral reactivations varied widely, from 7.5-62% of CMV reactivation, with the higher incidence exceeding the controls of this study (68, 70, 71, 75, 76). In two studies, EBV reactivation was higher than controls, again ranging widely among studies, from 4-71% with the lower incidence possibly linked to the addition of CD20 blockade in the regimens (70–73, 76). Deaths due to infection ranged from 15-17% overall (67, 74). Collectively, these data suggest that infectious immunity was relatively comparable, with the possible exception of CMV and EBV viral reactivations. The rates of graft failure were low (1-3%) with the exception of a single study (21%) (67–75). Overall survival was comparable in most studies, with a median of 64% (55-74%) and GRFS ranging from 38-50% (67–75). Collectively, these data support the use of rabbit ATG for the prevention of acute and chronic GVHD in adults undergoing HCT for hematologic malignancies, especially with peripheral blood donor source, with modest or no impact on GVL, engraftment, and most infections with the exception of viral reactivations.

## Post-transplant cyclophosphamide

Cyclophosphamide is an alkylating agent that causes DNA strand breakage and replication stress in dividing cells. Its use in the post-transplant setting to prevent GVHD was pioneered at Johns Hopkins University. It has been posited that post-transplant cyclophosphamide (PTCy) works through the preferential killing of alloreactive donor T cells followed by peripheral tolerance induction through clonal deletion and Treg suppression and lastly by central deletion of alloreactive T cell precursors in the thymus (77). However, our understanding of the mechanisms of action of PTCy is evolving and new evidence suggests that alloreactive T cells may not be eliminated but rather have reduced proliferation and impaired function (78). PTCy is typically paired with MMF and tacrolimus that begins following the last dose of cyclophosphamide. Waiting to start tacrolimus and MMF until after cyclophosphamide allows for unchecked alloreactive T cell proliferation in the absence of immunosuppression that is eliminated by the cyclophosphamide.

PTCy was first evaluated in non-myeloablative conditioning regimens for haploidentical transplants utilizing bone marrow grafts (79, 80). A dose of 50mg/kg of cyclophosphamide was given on days 3 and 4 post-transplant followed by MMF and tacrolimus on day +5 (79). The results of the Phase I/II trial revealed grade II-IV and grade III-IV aGVHD rates of 34% and 6% and extensive cGVHD of 5%. Graft rejection occurred in 13% of patients and the relapse rate at 2 years was 58% with a 2-year EFS of 26%. With the encouragingly low rates of GVHD in the initial trial, there have been numerous subsequent trials evaluating PTCy in the haploidentical setting with nonmyeloablative and reduced intensity conditioning. The median rates of grade II-IV aGVHD were 32% across the studies (ranging from 19-34%), 6% grade III-IV aGVHD (0-10%) and 26% cGVHD (13-34%) (79, 81–85). Relapse rates ranged from 31-58% with EFS of 26-55%. There was one randomized phase 3 study comparing double umbilical cord blood (dUCB) transplant to haploidentical with PTCy with reduced intensity conditioning which showed improved OS with haploidentical transplant but with higher rates of relapse. There was no difference in EFS, aGVHD, or CGVHD (83). With relapse being a concern in reduced intensity and nonmyeloablative haploidentical transplant with PTCy, more intensive conditioning has been evaluated (86–90). The median rates of grade II-IV aGVHD were 24% across the studies (ranging from 16-43%), 7% grade III-IV aGVHD (7-23%) and 30% cGVHD (15-56%). There appeared to be an improvement in relapse (22-44%), EFS (50-79%), and OS (67-85%) with the more intensive conditioning.

PTCy has also been evaluated in the matched related and matched and mismatched unrelated donor setting (81, 91–93). The median rates of grade II-IV aGVHD were 51% across the studies (ranging from 27-77%), 11% grade III-IV aGVHD (0-15%) and 16% cGVHD (9-28%) with the range of relapse, EFS, and OS being 17-28%, 60-69%, and 69-73%. The ACCESS study (NCT04904588) is an ongoing prospective Phase II study evaluating PTCy in HLA-mismatched unrelated donor transplant.

With the success of PTCy in the haploidentical and unrelated donor setting, there has been an increasing debate over whether a matched unrelated or haploidentical donor would be preferred given the lower cost and increased accessibility with a related donor. A recent CIBMTR analysis compared haploidentical and matched unrelated donor (MUD) transplants in which both groups received PTCy GVHD prophylaxis (84). In the reduced intensity setting, lower rates of grade II-IV aGVHD, grade III-IV aGVHD, and non-relapse mortality, while higher DFS and OS were observed in the matched unrelated donor (MUD) setting. No difference in cGVHD was seen. BMT CTN 1703, is a randomized, multicenter, phase III trial comparing PTCy, tacrolimus, and MMF to standard tacrolimus and methotrexate GVHD prophylaxis in patients receiving reduced intensity conditioning with a PBSC graft with hematologic malignancies. Donors will include matched siblings, matched and mismatched unrelated donors. The trial has completed enrollment however, data is not yet available.

The majority of the initial trials evaluating PTCy utilized bone marrow grafts but more recently PBSC grafts have been used. There have been no randomized prospective studies comparing graft sources however multiple retrospective studies have been performed with mixed results (94–97). While most studies have shown higher rates of aGVHD and/or cGVHD with PBSC grafts (95, 96, 98), survival differences have not been observed.

Delayed immune reconstitution and associated increased risk of infections has been observed with haploidentical transplant with PTCy (99–101). A recent CIBMTR analysis found higher rates of CMV reactivation with PTCy in the haploidentical and matched related setting compared to matched related with CNI based immunoprophylaxis (42%, 37%, and 23%, respectively) (99). In multivariate analysis, haploidentical patients receiving PTCy that were CMV-seropositive prior to transplant had significantly lower OS and higher NRM. Higher rates of non-CMV viral infections have also been reported with PTCy in haploidentical transplants (101).

PTCy has been less extensively studied in non-malignant disease and mostly limited to smaller studies. Low rates of GVHD and NRM have been observed however graft failure rates have been high with nonmyeloablative regimens (102–108). Improved engraftment rates have been seen with increased intensity of the conditioning (102). Larger studies evaluating PTCy in nonmalignant diseases are needed.

An additional consideration when using PTCy is the toxicities associated with cyclophosphamide. Cardiotoxicity is a known side effect of cyclophosphamide and tends to occur within the first few weeks following administration (109, 110). A recent study found a significantly higher incidence of early cardiac events with PTCy compared to other GVHD prophylactic regimens (109). Cardiotoxicity may also be exacerbated when using cyclophosphamide in the preparative regimen as higher cumulative doses have been associated with increased frequency of cardiac events (109, 110). Additional studies are needed to evaluate the frequency of cardiac toxicity in patients receiving PTCy.

In summary, PTCy has been evaluated in the haploidentical, matched-related, and the matched and mismatched unrelated donor setting and is associated with low rates of aGVHD and cGVHD. While PTCy has been associated with delayed immune reconstitution and increased risk of viral infections, the nonrelapse mortality rates are low. The impact on relapse and EFS is variable among studies but is improved in myeloablative and more HLA-matched transplants.

## Abatacept

For T cells to become activated they require both antigen presentation through T cell Receptor (TCR) engagement of the MHC on APCs and costimulation through multiple different costimulatory pathways (7). The best characterized costimulatory signal occurs between CD28 on T cells and CD80/86 on APCs (7). Abatacept is a fusion protein containing the extracellular domain of human CTLA4 and a modified Fc portion of IgG1. Abatacept can bind to CD80/86 and block T-cell costimulatory signaling and inhibit T cell activation (111). Abatacept was originally developed for use in rheumatoid arthritis and recently received FDA approval for the prevention of GVHD after allogeneic transplantation.

Based on encouraging data from an initial pilot study, one large prospective, multi-center, phase II, randomized, placebo-controlled, consortium study has been conducted to use abatacept to prevent aGVHD (112). Abatacept was added to a standard backbone of calcineurin inhibitor and methotrexate (on days -1, +5, +14, and +28) randomizing patients with hematologic malignancies with 8/8 HLA-matched unrelated donors to abatacept or placebo, with a singled-armed open label stratum of 7/8-HLA-mismated unrelated donors compared to a retrospective registry matched cohort (113). Abatacept exposure was associated with significantly lower grade II-IV aGVHD (43% for 8/8 cohort and 40% for the 7/8 cohort) and grade III-IV in the 7/8 cohort (2%). This reduction translated to improved severe aGVHD-free survival at day 180 (93% for the 8/8 cohort) and (100% for the 7/8 cohort compared to a registry cohort). Notably, this registry cohort of 7/8 patients had not received additional GVHD prophylaxis on the methotrexate and calcineurin inhibitor backbone. When ATG was added to the preparative regimen in an intention to treat analysis, the rate of II-IV aGVHD (40% with abatacept and 42% with ATG) was similar but grade III-IV aGVHD remained significantly higher (3% with abatacept and 22% with ATG) for the 7/8 cohorts. Despite the reduction in aGVHD, rates of cGVHD were similar with abatacept to controls, 62% overall for the 8/8 cohort and similar between abatacept and ATG for the 7/8 62%. The rates of moderate to severe cGVHD were also comparable for abatacept vs controls, 45% and 36% for the 8/8, not available for the 7/8 cohort. Abatacept did not increase relapse rates; 22% of abatacept-treated patients relapsed in the 8/8 cohort and 9% for the 7/8 cohort. The overall survival and event-free survival were similar in all groups, (74% 8/8+ abatacept vs. 80% 7/8+abatacept, and 66% 8/8+abatacept vs. 80% 7/8+abatacept respectively). The addition of abatacept appeared safe with similar engraftment (100% 7/8) and leukocyte reconstitution compared to controls. No significant increase in CMV or EBV reactivation or end-organ disease was observed, however there was trend towards increased CMV reactivation with abatacept compared to placebo (47% vs 33%). Abatacept has also been evaluated in smaller studies of patients with non-malignant diseases (114) with encouraging results. Larger studies are currently ongoing and include extended dosing of abatacept, (NCT03924401, NCT02867800, NCT04380740). In summary, abatacept effectively reduced rates of acute GVHD, tested in 7/8 and 8/8 HLA-matched donors for children and adults with hematologic malignancies, without impairing leukemia control nor engraftment, not seeming to increase infectious risk, with comparable rates of cGVHD in a large prospective randomized study with more in progress at this time.

## CD34+ selection

In contrast to *in vivo* approaches to reduce GVHD, *ex vivo* approaches utilize graft engineering to modify the product prior to infusion. CD34+ positive selection was one of the first such approaches, which the goal of a product highly enriched for CD34+ stem cells with minimal CD3+ lymphocytes, administered on a backbone of ATG (usually rabbit though some studies included equine) without subsequent pharmacologic immunosuppression. The CD34+ selection strategy has largely been used in adult recipients of matched related or unrelated donors for the treatment of hematologic malignancy. CD34+ cell doses are a median of 6-8e6/kg with T cell doses infused of 2-6e6/kg (115–117). Rates of acute GVHD were low, median 16% (range 11-23), with 19% chronic GVHD (3-19%) (74, 115–117). Relapse rates were comparable to other approaches ranging from 22-29% (74, 115–117). While graft failure was only reported in 1 study in 1 patient (of 712 patients) (74, 115–117). Overall survival ranged from 57-70% and only one study reported a GRFS of 59% (74, 115–117). Not surprisingly, infections were commonly reported, observed in 73%-77% of patients, with 57-61% bacterial, 11% invasive fungal, and one study reporting 43% and 18% CMV and EBV reactivations respectively (116, 117). Infections complicated 42-57% of deaths (74, 115). Two small studies have been conducted in pediatrics, totaling 28 patients, approximately half of whom were treated for hematologic malignancies, and all received haploidentical donors (118, 119). While these studies included higher stem cell dose (19 and 21.5 x10e6) and higher T cell doses (1.4 and 4.7 x10e4), the rates of graft failure were high at 21 and 25%, with few patients eligible for relapse (1/10) but low rates of aGVHD and cGVHD, ~16% and 0 and 24% respectively (118, 119). Collectively, these data supported the benefit of CD34+ selection for reduction of acute and chronic GVHD, albeit with high rates of infection for adults with hematological malignancies and graft failure in pediatrics, with no data for alternative donors, minimal for pediatric recipients, and other transplant indications.

## CD45RA depletion

Murine data initially demonstrated that the alloreactive T cells that engender GVHD are found within the naïve T cell populations (15, 120–122). This led to a graft engineering approach that selectively depleted CD45RA naïve T cells, preserving the CD34+ fraction critical for engraftment and CD45RO memory cells that could maintain T cell activity against infections and tumors (123). Studies in human cells validated that CD8+ T cells reactive to minor H antigens were 5-20 fold higher in the naïve rather than memory fraction (124). Three studies have utilized this approach. In two, which include an initial phase II and then a larger follow-on study (n=138), the preparative regimen for adult recipients of matched sibling transplants for acute leukemia was modified to remove the standard methotrexate and replace it with this targeted approach of naïve T cell reduction. While the incidence of cGVHD was only 7 and 9%, the incidence of grade II-IV aGVHD was 66% and 75%, albeit largely steroid responsive (125, 126). Rates of relapse (21 and 23%) and overall survival (77 and 78%) were similar to other approaches and without graft failure (n=35) (125, 126). Another group employed this approach in haplo-identical transplant for children with acute leukemia, using sirolimus and MMF for additional GVHD control, CD20 depletion, and adoptive transfer of NK cells post-HCT for disease control (127). In this platform, the rates of grade II-IV aGVHD were modest (18%), but the rates of cGVHD were already 35% with short follow-up (~100 days) (127). Notably, while late outcomes have not been reported in full, a follow-on paper did report a high incidence of HHV6 reactivations with this approach (128). In summary, this is an intriguing approach to selectively modify the T cell fraction of the graft with data to support low rates of cGVHD in the matched donor setting, though with higher rates of aGVHD, largely tested in the pediatric setting.

## Alpha/beta T cell/B cell depletion (αßTB depletion)

Another ex-vivo graft engineering approach includes the depletion of alpha/beta T cells and B cells targeting higher CD34+ doses (~10-15x10e6 CD34+ cells/kg) obtained *via* peripheral blood stem cell collection. Most of these protocols employ anti-thymocyte globulin and rituximab (CD20+ B cell depletion) as part of the transplant regimen, with the former aimed to decrease the risks of graft rejection and GVHD and the latter to decrease EBV reactivation. Most of these protocols do not employ post-transplant pharmacologic GVHD prophylaxis, testing whether the graft engineering approach is sufficient to avert alloimmunity alone. This *αßTB depletion* approach was developed to increase donor accessibility with haplo-identical transplantation, removing the cells most likely to induce GVHD, while preserving graft-vs-leukemia and anti-infectious immunity through preservation of natural killer and gamma-delta T cells. Thus, most but not all the studies use this graft engineering approach with related HLA-mismatched donors. This newer therapy has largely been studied in children, comprising 95% of the nearly 700 total patients reported in the literature, and a still small cohort compared to other alternative donor approaches (129–141). More data is needed to evaluate the success of this approach in adult HCT recipients and would be beneficial in pediatric cohorts as well.

Because *αßTB depletion* removes the T cells most closely linked to acute and chronic GVHD and depletes the B cells that can contribute to chronic GVHD, the rates of these complications were anticipated to be lower than that observed after matched donor HCTs despite the increased HLA-mismatch. Overall, this has been observed with median rates of grade II-IV aGVHD of 18% (ranging from 11-28%), and cGVHD of 8% (range 0-30%), with 0-21% experiencing extensive cGVHD (129–144). Notably, most of these studies utilized ATG, which has reduced aGVHD (see above), and not surprisingly, in one study in which ATG was removed, the observed rates of aGVHD were increased (136). The relapse rates ranged from 18-30% for those utilizing *αßTB depleted* HCT for the treatment of hematologic malignancies, comparable overall to other approaches (129, 135–137, 141, 143, 144). These data are confounded by a difference in the conditioning regimen; most have included myeloablative total body irradiation for all hematologic malignancies, a difference from other regimens that could potentially diminish relapse through greater leukemia clearance (129, 136, 137, 141). One study of *αßTB depleted* HCT included matched unrelated donors and removed ATG from this preparative regimen due to increased relapsed rates with subsequent associated higher rates of aGVHD, suggesting that this preparative regimen may be best used in the haplo-identical setting (136). This approach has been used for the treatment of relapsed refractory acute myeloid leukemias, without ATG, substituting tocilizumab therapy (anti-IL6), and scheduled CD45ra immature T cell depleted infusions with or without hypomethylating therapy. While small numbers (n=25), an initial complete remission was observed in 95%, and while 42% relapsed, the 3 year event-free survival was 49%,with only 18% aGHVD and 23% cGVHD, suggesting that this approach might have good anti-leukemia control as these numbers exceed historical controls (145). Two studies incorporated zoledronic acid to enhance gamma/delta T cell activity with a trend toward improvement in event-free survival (129, 146). Collectively, these data support that the *αßTB depleted haplo-*HCT approach may have potent anti-leukemia potential though the optimal additional agents (Tocilizumab vs. ATG or zoledronic acid) have yet to be established. As expected with *αß*TB cell depletion, the rates of viral reactivation were higher than matched transplant approaches. Those reporting viral reactivations reported consistent rates of 40-65% of patients experiencing reactivations, with 20-65% reactivating CMV, 0-44% EBV, up to 57% adenovirus, 21% HHV6, and 23% BK virus (129–134, 136–142). A large retrospective review showed the incidence of CMV and EBV reactivation were 53% and 33% respectively after *αß*TB cell depletion and associated with aGVHD (147). While rituximab use pre-transplant does appear to reduce the risk of EBV reactivation, these data show that it can still occur (as most of these group did use rituximab), though the rates of EBV post-transplant lymphoproliferative disease were quite low, at 0.5%, suggesting that this is a treatable problem for most patients (147).

While graft failure was low in patients receiving *αßTB depletion* for malignant indications (0-3%), the incidence was higher in nonmalignant recipients, ranging from 4-30%, and appears higher than the rates of graft failure rates reported in a large CIBTMR study of similar pediatric populations (129–142, 144). The overall survival in pediatric hematologic malignant patients was 67-90%, 54% for adult, and 84-100% for non-malignant (129–142). The GRFS in pediatric patients was 50%-61% in hematologic malignancy patients and was 69-87% in the pediatric non-malignant cohort (129–142). In summary, this approach is best studied in pediatric HCT recipients with malignant and nonmalignant diseases, with good anti-leukemia effect, with particular attention paid to the risks of viral reactivations, which should include frequent viral infection surveillance and the plan for preventative or pre-emptive approaches.

## Cord blood

Umbilical cord blood (UCB) was first identified as a viable alternative donor source in 1989 (148), permitting the use of mismatched transplant with acceptable rates of GVHD. Despite lower total nucleated cell and CD34+ cell counts, UCB has been found to have a higher proportion of hematopoietic progenitor cells than BM and PBSC grafts as well as greater proliferation potential of CD34+ progenitors (149–152). The initial use of UCB grafts was restricted to pediatric patients with related donors due to concerns about low cell doses in adults and the lack of UCB banks (153). however this has now been overcome with the use of double umbilical cord blood and studies are underway to evaluate other modes of hematopoietic progenitor cell expansion as well (154, 155).

One advantage of UCB transplant has been its success even in the setting of HLA disparity. Due to lower immunogenicity, UCB transplant requires less stringent HLA-matching (out of 6 rather than the 8 typically used for adult donor matching). Greater HLA disparity has been associated with increased rates of graft failure and GVHD (154, 156–158) and studies in non-malignant disease have shown higher rates of rejection and delayed immune reconstitution which is improved with higher cell doses and better matching (159, 160).

Large studies evaluating single and double UCB transplant have reported median rates of grade II-IV aGVHD of 38% (ranging from 24-45%), 17% grade III-IV aGVHD (9-25%) and 27% cGVHD (14-53%) with the range of relapse, EFS, and OS being 12-45%, 28-70%, and 31-75% respectively (14, 55, 161–170). Some studies comparing single and double UCB transplants have shown increased rates of aGVHD and/or cGVHD (169, 170), while others have found no differences (14, 55, 167, 168). Lower rates of cGVHD have also been observed with UCB when compared to matched related or unrelated donors (162, 164–166). Several studies have also suggested potential for increased graft-versus-leukemia effect and lower relapse with UCB transplant compared to matched related and unrelated BM and PBSC grafts (154, 165, 170–172). The incidence of NRM in UCB transplant is highly variable (6-42%) (14, 24, 123–132) depending on the study. In addition to higher rates of rejection, delayed immune reconstitution and consequently higher infection rates (particularly viral infections) are frequently reported with UCB (164, 173–176). The higher infection rate is thought to be related to the delayed recovery of naïve and memory T cells that is observed with UCB (173) and the absence of passive transference of humoral immunity from the donor. The use of ATG in the conditioning regimen has also been linked to higher NRM (63, 64, 177, 178). The timing of ATG in proximity to the graft infusion may play a role in poorer outcomes (178).

In summary, UCB transplant is associated with tolerable rates of grade II-IV aGVHD and reduced rates of cGVHD while permitting greater HLA disparity, though mismatched donors have been associated with higher GVHD and NRM. Delayed immune reconstitution and higher infection rates and their effect on NRM are a concern with UCB transplant while the graft versus leukemia effect appears to be preserved.

## Experimental approaches

The prevention of graft versus host disease after allogeneic hematopoietic cell transplantation is a rapidly evolving field. In addition to the strategies listed above, many are currently in development and/or clinical trials. A clinicaltrials.gov search yielded over 50 studies currently active, including engineered grafts that supplement immunomodulatory cells e.g. Treg cells (“Orca-T”) NCT03802695 or interferon gamma-primed mesenchymal stromal cells NCT04328714, *in vivo* depletion with Obinutuzumab (CD20 blockade) NCT02867384, ustekinumab (IL-12 and IL-23 blockade) NCT04572815, tildrakizumab (IL-23 blockade) NCT04112810, or BAFF inhibition with belimumab NCT03207958, or the addition of medications that modulate the immune system, e.g. proteasome inhibition with Ixazomib NCT03225417, histone deacetylase inhibition with vorinostat NCT03842696, JAK1 inhibition with itacitiniab NCT04859946, or the addition of alpha-1-antitrypsin NCT03805789.

## Controversies in GVHD prevention strategies

### What regimens might prioritize reduction of acute or chronic GVHD or both?

While aGVHD is a risk factor for cGVHD, not all regimens that reduce one reduce the other. The early data for naïve T cell depletion does not appear to impact rates of aGVHD, especially as this was tested in patients with matched sibling donors and thus those at the lowest risk for GVHD. That said, if one develops highly treatable aGVHD with low rates of cGVHD and relapse, would this be the method of choice for patients with very high-risk hematologic malignancies and a matched sibling donor? Notably, this was tested in young adults and evaluation in pediatrics is underway but unknown. In addition, there is no data for nonmalignant indications, and for these patients neither aGVHD nor cGVHD provides benefit (in contrast to the potential benefit of concurrent graft versus leukemia effect, GVL). ATG may reduce both but is this needed for all matched sibling transplants? This may be an especially critical query for young children who incur low rates of chronic GVHD and may have aggressive malignant disease with higher relapse rates. Abatacept has also been tested in children and adults with matched and mismatched unrelated donors and has reduced the rates of severe aGVHD in those with high-risk hematologic malignancies but with the same query for 8/8 matched transplants. Similarly, should CD34 selection be chosen for these patients even if it reduces the rates of acute and chronic GVHD? Should the age of the recipient and/or donor influence the choice of additional prophylaxis? Perhaps, one should consider late teens to older adults a population to consider for these trials in the future?

While this remains an ongoing question in the matched transplant donor setting, historical data clearly justify the use of additional GVHD prophylaxis for alternative donors (<8/8 match), in which unacceptable rates of acute and/or chronic GVHD have been demonstrated. For those without an 8/8 matched donor, the choices typically include a 7/8 matched unrelated donor, a haplo-identical donor, and cord blood transplantation. For 7/8 matched unrelated donors, abatacept maintained good antileukemia control, with event free survival rates matching matched cohorts, albeit with some increase in cGVHD. The dose of abatacept is now being extended to evaluate if both acute and chronic GVHD may be averted by this method. However, on balance, good leukemia control may be more valuable with high-risk diseases and merit a higher risk of cGVHD, and this approach did extend the donor pool for many patients by including mismatched unrelated donors. ATG impacted aGVHD and cGVHD in this setting as well, though some studies suggested an impact on graft versus leukemia effects. Alternatively, in the haplo-identical setting, PTCy reduced aGVHD and cGVHD, albeit with some increased in viral reactivations in both approaches and some studies showing higher relapse and nonengraftment. Similarly, in children, the haplo-identical platform has included *αß*TB depletion, which reduced cGVHD without aGHVD, with similar risks of graft failure and viral reactivations though as yet to be studied in adults. Additionally, cord blood reduces the risk of cGVHD, especially in matched settings, while still incurring a risk of aGVHD but potentially preserving GVL. Abatacept, ATG, cord blood, *αß*TB depletion, and PTCy markedly increase the donor pool to include mismatched donors without severe acute and/or chronic GVHD, critical as many HCT recipients lack matched donors. Collectively, these data support a personalized approach to the risks of acute and chronic GVHD, including thoughtful evaluation of the risk of relapse, the risk of severe acute or chronic GVHD (e.g., based on age, intensity of preparative regimen), the risk of nonengraftment, and the risks of severe infections.

It is noteworthy that there is large variability in the number of pediatric and adult patients in whom each approach has been tested. The largest data exists for ATG, cord, and PTCy with each of these including some prospective comparison studies with less data for the other (often newer) approaches. In addition, a limitation to comparing approaches is that not all studies report acute and chronic GVHD incidence, infections, graft failure, relapse rates, and GRFS. As we move forward, it would be helpful for each trial to present complete data on these variables to aid in precision medicine of GVHD prophylaxis and to prioritize prospective comparison trials.

### What is the best regimen for patients at higher risk for graft failure?

Certain indications for transplant incur higher risks of nonengraftment, due to either decreased fertile stromal milieu (e.g., myelofibrosis) or activated host T cell populations (e.g., autoimmune diseases). When choosing GVHD prophylaxis regimens for these patients, the risks of nonengraftment should be prioritized. Given that PTCy, *αß*TB depletion, and cord blood may be potentially associated with increased risks of nonengraftment compared to other approaches, these approaches might be deprioritized in these patients. Further, one might also consider the impact of residual ATG on these patients, adopting a pharmacokinetic approach to mitigate the effect on donor T cells that can aid in engraftment. Alternatively, there may be other ways to mitigate this risk, such as testing for donor specific antibodies and using this for donor selection, agents that could enhance the stromal function, or targeting higher stem cell doses either by increased volume at collection or by CD34 stem cell expansion strategies.

### What is the best regimen for those at highest risk for relapse of malignant disease?

The regimen that best preserves the graft versus leukemia effect remains to be determined. In part, this is because the key lymphocytes and tumor milieu that mediate GVL remain elusive. Data support that functional lymphocytes are key to maintain GVL (179). Thus, GVHD prevention strategies for malignant disease are challenged by competing interests of T cell immunity against infections and the need to maintain active GVL, while desiring less T cell activity to achieve tolerance, especially in the mismatched transplant setting. As relapse remains the primary cause of death after HCT for malignant indications, this is of paramount importance. Several studies have linked cord blood to lower rates of relapse but not all. Higher doses of ATG were linked to relapse in several studies, though lower doses did not appear to increase relapse rates and strategies to optimize timing and dose based on lymphocyte count may be valuable to optimize this strategy. Some studies had higher rates of relapse in other regimens, though not born out by others. Variability of disease status at the time of transplant may have contributed to some of these data, with some studies permitting residual disease (e.g., for myeloid diseases) and others largely enrolling those without evidence of disease, and de facto, decreasing the risks of relapse.

It is also important to consider other factors that alter the risk of relapse. Reduced preparative regimens increase relapse risk, though are necessary for those with diminished organ function or advanced age. Alternatively, some approaches rely upon total body irradiation which may increase the anti-leukemia benefit by disease reduction in sites with diminished blood flow or high residence in the marrow stromal niches. An increased risk of relapse after HCT may be mitigated by post-transplant disease reduction approaches as well, *via* pharmacologic interventions (e.g., hypomethylating agents, FLT3 blockade) or *via* adoptive transfer of donor lymphocytes. These donor lymphocytes infusions have historically been unmanipulated, though newer approaches include removal of CD45RA naïve T cells, or manipulation to expand T cells specific for tumor antigens. Collectively, these considerations suggest that these factors should be carefully considered as the preparative and GVHD prophylaxis regimens are developed, including optimization of initial disease reduction, consideration of the effects of graft versus tumor effect on the GVHD prophylaxis chosen, and the post-transplant options for maintenance of remission.

### What are the GVHD prophylaxis considerations for those with nonmalignant disease?

Many patients who undergo transplant for nonmalignant diseases have increased risk for infections (e.g., primary immunodeficiency), non-engraftment, or increased risk for particular organ impairments (e.g., pulmonary and renal dysfunction from sickle cell disease). Those who enter HCT with active viral infections in the setting of pre-existing T cell dysfunction may benefit from optimizing early T cell function to avert life-threatening viral disease. In this case, abatacept and ATG may be better than PTCy, CD34+ selection, αßTB depleted haplo, or cord blood in which the viral reactivations may be higher. Alternatively, other supportive care approaches may also be employed to mitigate this risk. Letermovir is now approved for prophylaxis of CMV in adult HCT recipients at high risk of reactivation and thus diminish this risk. Similarly, the planned use of T cells specific for particular viruses (through selective expansion or cytokine capture) may also enable more donor choices through decreasing these viral risks.

In contrast, those with sickle cell disease who are less likely to have matched donors may benefit from PTCy or abatacept to increase the donor pool without increasing adverse effects (to the kidney and lungs). Some of these patients with nonmalignant disease may require rapid transplantation which may prioritize the PTCy or *αß*TB depletion, permitting expedited HCT with mismatched related donor, both accessible and likely motivated to proceed.

It is also notable that several groups are developing preparative regimens without cytotoxic chemotherapy. This may shift the landscape for these patients, potentially mitigating aGVHD risk, and permitting prioritization of reduction of cGVHD for these patients.

Finally, there are a dearth of data regarding GVHD prophylaxis to treat nonmalignancy. This is surprising as this population may be the ideal population to test various prophylaxis options. Relapse is not a competing risk and estimates suggest ~25% of patients will develop cGVHD (144).

### What is the optimal regimen for immune reconstitution?

The approaches to prevent GVHD, including donor sources, have varying degrees of impact on immune reconstitution. Delayed immune reconstitution is linked to increased infectious complications and associated mortality. Some patients have baseline infectious risk factors necessitating a more rapid immune recovery, e.g., neutrophil recovery for active fungal disease or high-risk bacterial infections or T cell recovery for a history of mycobacterial infections or viral reactivations or active infections. Cord blood has been associated with delayed T cell reconstitution and infections compared to adult donors, attributed to the absence of memory cells to effect viral immunity. Additionally, PBSC grafts have been found to have significantly quicker neutrophil recovery than BM grafts. PTCy has also been linked to delayed T cell reconstitution and increased risk of viral infections, while abatacept showed similar immune reconstitution to controls.

Full T cell reconstitution requires two pathways for recovery. Initially, the infused donor T cells will increase number but not diversity through peripheral expansion. These initial T cells can provide GVL and viral immunosurveillance. Full recovery of diverse T cell reconstitution requires thymopoiesis, an option for children and young adults after hematopoietic cell transplantation. Little is known about the thymic recovery of the different GVHD prophylaxis approaches. However, minimizing the risk of GVHD overall should diminish one risk of thymus impairment, that of direct T cell attack on the thymus tissue. Including studies of thymus-derived T cells, diversity of T cell receptor, evidence of T cell function (e.g., vaccine response), would be beneficial to evaluate the overall effect of each approach on lymphoid immune reconstitution.

## Summary

In conclusion, the approach to acute and chronic GVHD prophylaxis should be personalized, factoring in the age of the recipient (and potential donor), the preexisting conditions and disease indication, the intensity of the conditioning regimen, the risk of, and opportunities to mitigate infections or disease relapse/recurrence, and the risks of nonengraftment and delayed immune reconstitution. Recently, there have been many new approaches that have both diminished the rates of severe acute and/or chronic GVHD, that have increased both the available donor pool and the options for personalized GVHD prophylaxis regimens. Comparisons of alternative donor approaches would potentially be valuable for particular disease indications across the lifetime spectrum of the recipient, to assist practitioners in these weighty decisions. In addition, the optimal GVHD prophylaxis is influenced by other aspects of the HCT, e.g., preparative regimen intensity, enhanced supportive care for the diagnosis and treatment of infections, which may reprioritize the prophylaxis regimens for particular HCT recipients.

## Author contributions

BW and KW designed, wrote, and edited the manuscript. All authors contributed to the article and approved the submitted version.

## Conflict of interest

The authors declare that the research was conducted in the absence of any commercial or financial relationships that could be construed as a potential conflict of interest.

## Publisher’s note

All claims expressed in this article are solely those of the authors and do not necessarily represent those of their affiliated organizations, or those of the publisher, the editors and the reviewers. Any product that may be evaluated in this article, or claim that may be made by its manufacturer, is not guaranteed or endorsed by the publisher.

## References

[1] Arai S Jagasia M Storer B Chai X Pidala J Cutler C. Global and organ-specific chronic graft-versus-host disease severity according to the 2005 NIH consensus criteria. Blood (2011) 118:4242–9. doi: 10.1182/blood-2011-03-344390 PMC3204740 21791424

[2] Couriel DR Saliba RM Giralt S Khouri I Andersson B de Lima M. Acute and chronic graft-versus-host disease after ablative and nonmyeloablative conditioning for allogeneic hematopoietic transplantation. Biol Blood Marrow Transplant (2004) 10:178–85. doi: 10.1016/j.bbmt.2003.10.006 14993883

[3] Blazar BR Murphy WJ Abedi M. Advances in graft-versus-host disease biology and therapy. Nat Rev Immunol (2012) 12:443–58. doi: 10.1038/nri3212 PMC3552454 22576252

[4] Choi SW Levine JE Ferrara JL. Pathogenesis and management of graft-versus-host disease. Immunol Allergy Clin North Am (2010) 30:75–101. doi: 10.1016/j.iac.2009.10.001 20113888 PMC4141413

[5] Hill GR Crawford JM Cooke KR Brinson YS Pan L Ferrara JL. Total body irradiation and acute graft-versus-host disease: The role of gastrointestinal damage and inflammatory cytokines. Blood (1997) 90:3204–13. doi: 10.1182/blood.V90.8.3204 9376604

[6] Penack O Holler E van den Brink MR. Graft-versus-host disease: Regulation by microbe-associated molecules and innate immune receptors. Blood (2010) 115:1865–72. doi: 10.1182/blood-2009-09-242784 20042727

[7] Smith-Garvin JE Koretzky GA Jordan MS. T Cell activation. Annu Rev Immunol (2009) 27:591–619. doi: 10.1146/annurev.immunol.021908.132706 19132916 PMC2740335

[8] Xing Y Hogquist KA. T-Cell tolerance: central and peripheral. Cold Spring Harb Perspect Biol (2012) 4:1-15. doi: 10.1101/cshperspect.a006957 PMC3367546 22661634

[9] Newton-Nash DK. The molecular basis of allorecognition. assessment of the involvement of peptide. Hum Immunol (1994) 41:105–11. doi: 10.1016/0198-8859(94)90002-7 7860354

[10] Matte-Martone C Liu J Jain D McNiff J Shlomchik WD. CD8+ but not CD4+ T cells require cognate interactions with target tissues to mediate GVHD across only minor h antigens, whereas both CD4+ and CD8+ T cells require direct leukemic contact to mediate GVL. Blood (2008) 111:3884–92. doi: 10.1182/blood-2007-11-125294 PMC2275040 18223170

[11] Nikolic B Lee S Bronson RT Grusby MJ Sykes M. Th1 and Th2 mediate acute graft-versus-host disease, each with distinct end-organ targets. J Clin Invest (2000) 105:1289–98. doi: 10.1172/JCI7894 PMC315439 10792004

[12] Jiang H Fu D Bidgoli A Paczesny S. T Cell subsets in graft versus host disease and graft versus tumor. Front Immunol (2021) 12:761448. doi: 10.3389/fimmu.2021.761448 34675938 PMC8525316

[13] Divito SJ Aasebo AT Matos TR Hsieh PC Collin M Elco CP. Peripheral host T cells survive hematopoietic stem cell transplantation and promote graft-versus-host disease. J Clin Invest (2020) 130:4624–36. doi: 10.1172/JCI129965 PMC7456221 32516138

[14] Zheng H Matte-Martone C Li H Anderson BE Venketesan S Sheng Tan H. Effector memory CD4+ T cells mediate graft-versus-leukemia without inducing graft-versus-host disease. Blood (2008) 111:2476–84. doi: 10.1182/blood-2007-08-109678 PMC2234071 18045967

[15] Anderson BE McNiff J Yan J Doyle H Mamula M Shlomchik MJ. Memory CD4+ T cells do not induce graft-versus-host disease. J Clin Invest (2003) 112:101–8. doi: 10.1172/JCI17601 PMC162285 12840064

[16] Dutt S Tseng D Ermann J George TI Liu YP Davis CR. Naive and memory T cells induce different types of graft-versus-host disease. J Immunol (2007) 179:6547–54. doi: 10.4049/jimmunol.179.10.6547 17982043

[17] Flowers ME Inamoto Y Carpenter PA Lee SJ Kiem HP Petersdorf EW. Comparative analysis of risk factors for acute graft-versus-host disease and for chronic graft-versus-host disease according to national institutes of health consensus criteria. Blood (2011) 117:3214–9. doi: 10.1182/blood-2010-08-302109 PMC3062319 21263156

[18] Hahn T McCarthy PL Jr. Zhang MJ Wang D Arora M Frangoul H. Risk factors for acute graft-versus-host disease after human leukocyte antigen-identical sibling transplants for adults with leukemia. J Clin Oncol (2008) 26:5728–34. doi: 10.1200/JCO.2008.17.6545 PMC2645611 18981462

[19] Watkins BK Horan J Storer B Martin PJ Carpenter PA Flowers ME. Recipient and donor age impact the risk of developing chronic GvHD in children after allogeneic hematopoietic transplant. Bone Marrow Transplant (2017) 52:625–6. doi: 10.1038/bmt.2016.328 27991888

[20] Golob JL Pergam SA Srinivasan S Fiedler TL Liu C Garcia K. Stool microbiota at neutrophil recovery is predictive for severe acute graft vs host disease after hematopoietic cell transplantation. Clin Infect Dis (2017) 65:1984–91. doi: 10.1093/cid/cix699 PMC5850019 29020185

[21] Han L Jin H Zhou L Zhang X Fan Z Dai M. Intestinal microbiota at engraftment influence acute graft-Versus-Host disease *via* the Treg/Th17 balance in allo-HSCT recipients. Front Immunol (2018) 9:669. doi: 10.3389/fimmu.2018.00669 29740427 PMC5928130

[22] Peled JU Gomes ALC Devlin SM Littmann ER Taur Y Sung AD. Microbiota as predictor of mortality in allogeneic hematopoietic-cell transplantation. N Engl J Med (2020) 382:822–34. doi: 10.1056/NEJMoa1900623 PMC7534690 32101664

[23] Williams KM Inamoto Y Im A Hamilton B Koreth J Arora M. National institutes of health consensus development project on criteria for clinical trials in chronic graft-versus-Host disease: I. the 2020 etiology and prevention working group report. Transplant Cell Ther (2021) 27:452–66. doi: 10.1016/j.jtct.2021.02.035 PMC8217141 33877965

[24] Zhang Q Raoof M Chen Y Sumi Y Sursal T Junger W. Circulating mitochondrial DAMPs cause inflammatory responses to injury. Nature (2010) 464:104–7. doi: 10.1038/nature08780 PMC2843437 20203610

[25] Anasetti C Logan BR Lee SJ Waller EK Weisdorf DJ Wingard JR. Peripheral-blood stem cells versus bone marrow from unrelated donors. N Engl J Med (2012) 367:1487–96. doi: 10.1056/NEJMoa1203517 PMC3816375 23075175

[26] Arai S Arora M Wang T Spellman SR He W Couriel DR. Increasing incidence of chronic graft-versus-host disease in allogeneic transplantation: A report from the center for international blood and marrow transplant research. Biol Blood Marrow Transplant (2015) 21:266–74. doi: 10.1016/j.bbmt.2014.10.021 PMC4326247 25445023

[27] Arora M Cutler CS Jagasia MH Pidala J Chai X Martin PJ. Late acute and chronic graft-versus-Host disease after allogeneic hematopoietic cell transplantation. Biol Blood Marrow Transplant (2016) 22:449–55. doi: 10.1016/j.bbmt.2015.10.018 PMC4787270 26541363

[28] Baird K Cooke K Schultz KR. Chronic graft-versus-host disease (GVHD) in children. Pediatr Clin North Am (2010) 57:297–322. doi: 10.1016/j.pcl.2009.11.003 20307722 PMC2872081

[29] Cuvelier GDE Nemecek ER Wahlstrom JT Kitko CL Lewis VA Schechter T. Benefits and challenges with diagnosing chronic and late acute GVHD in children using the NIH consensus criteria. Blood (2019) 134:304–16. doi: 10.1182/blood.2019000216 PMC6911839 31043425

[30] Zecca M Prete A Rondelli R Lanino E Balduzzi A Messina C. Chronic graft-versus-host disease in children: incidence, risk factors, and impact on outcome. Blood (2002) 100:1192–200. doi: 10.1182/blood-2001-11-0059 12149197

[31] Brunstein CG Gutman JA Weisdorf DJ Woolfrey AE Defor TE Gooley TA. Allogeneic hematopoietic cell transplantation for hematologic malignancy: relative risks and benefits of double umbilical cord blood. Blood (2010) 116:4693–9. doi: 10.1182/blood-2010-05-285304 PMC2996124 20686119

[32] Brunstein CG Miller JS McKenna DH Hippen KL DeFor TE Sumstad D. Umbilical cord blood-derived T regulatory cells to prevent GVHD: kinetics, toxicity profile, and clinical effect. Blood (2016) 127:1044–51. doi: 10.1182/blood-2015-06-653667 PMC4768428 26563133

[33] Marks DI Woo KA Zhong X Appelbaum FR Bachanova V Barker JN. Unrelated umbilical cord blood transplant for adult acute lymphoblastic leukemia in first and second complete remission: A comparison with allografts from adult unrelated donors. Haematologica (2014) 99:322–8. doi: 10.3324/haematol.2013.094193 PMC3912963 24056817

[34] Peffault de Latour R Chevret S Jubert C Sirvent A Galambrun C Ruggeri A. Unrelated cord blood transplantation in patients with idiopathic refractory severe aplastic anemia: a nationwide phase 2 study. Blood (2018) 132:750–4. doi: 10.1182/blood-2018-01-829630 29760162

[35] Matsuoka K Kim HT McDonough S Bascug G Warshauer B Koreth J. Altered regulatory T cell homeostasis in patients with CD4+ lymphopenia following allogeneic hematopoietic stem cell transplantation. J Clin Invest (2010) 120:1479–93. doi: 10.1172/JCI41072 PMC2860902 20389017

[36] Zorn E Kim HT Lee SJ Floyd BH Litsa D Arumugarajah S. Reduced frequency of FOXP3+ CD4+CD25+ regulatory T cells in patients with chronic graft-versus-host disease. Blood (2005) 106:2903–11. doi: 10.1182/blood-2005-03-1257 PMC1895303 15972448

[37] Du J Paz K Thangavelu G Schneidawind D Baker J Flynn R. Invariant natural killer T cells ameliorate murine chronic GVHD by expanding donor regulatory T cells. Blood (2017) 129:3121–5. doi: 10.1182/blood-2016-11-752444 PMC5465838 28416503

[38] Kolupaev OV Dant TA Bommiasamy H Bruce DW Fowler KA Tilley SL. Impaired bone marrow b-cell development in mice with a bronchiolitis obliterans model of cGVHD. Blood Adv (2018) 2:2307–19. doi: 10.1182/bloodadvances.2017014977 PMC6156893 30228128

[39] Pierini A Nishikii H Baker J Kimura T Kwon HS Pan Y. Foxp3(+) regulatory T cells maintain the bone marrow microenvironment for b cell lymphopoiesis. Nat Commun (2017) 8:15068. doi: 10.1038/ncomms15068 28485401 PMC5436085

[40] Radojcic V Paz K Chung J Du J Perkey ET Flynn R. Notch signaling mediated by delta-like ligands 1 and 4 controls the pathogenesis of chronic GVHD in mice. Blood (2018) 132:2188–200. doi: 10.1182/blood-2018-03-841155 PMC6238189 30181175

[41] Srinivasan M Flynn R Price A Ranger A Browning JL Taylor PA. Donor b-cell alloantibody deposition and germinal center formation are required for the development of murine chronic GVHD and bronchiolitis obliterans. Blood (2012) 119:1570–80. doi: 10.1182/blood-2011-07-364414 PMC3286218 22072556

[42] Deng R Hurtz C Song Q Yue C Xiao G Yu H. Extrafollicular CD4(+) T-b interactions are sufficient for inducing autoimmune-like chronic graft-versus-host disease. Nat Commun (2017) 8:978. doi: 10.1038/s41467-017-00880-2 29042531 PMC5645449

[43] Jin H Ni X Deng R Song Q Young J Cassady K. Antibodies from donor b cells perpetuate cutaneous chronic graft-versus-host disease in mice. Blood (2016) 127:2249–60. doi: 10.1182/blood-2015-09-668145 PMC4859199 26884373

[44] Zhang C Todorov I Zhang Z Liu Y Kandeel F Forman S. Donor CD4+ T and b cells in transplants induce chronic graft-versus-host disease with autoimmune manifestations. Blood (2006) 107:2993–3001. doi: 10.1182/blood-2005-09-3623 16352808

[45] Arai S Sahaf B Narasimhan B Chen GL Jones CD Lowsky R. Prophylactic rituximab after allogeneic transplantation decreases b-cell alloimmunity with low chronic GVHD incidence. Blood (2012) 119:6145–54. doi: 10.1182/blood-2011-12-395970 PMC3383022 22563089

[46] Cutler C Kim HT Bindra B Sarantopoulos S Ho VT Chen YB. Rituximab prophylaxis prevents corticosteroid-requiring chronic GVHD after allogeneic peripheral blood stem cell transplantation: results of a phase 2 trial. Blood (2013) 122:1510–7. doi: 10.1182/blood-2013-04-495895 PMC3750344 23861248

[47] Alexander KA Flynn R Lineburg KE Kuns RD Teal BE Olver SD. CSF-1-dependant donor-derived macrophages mediate chronic graft-versus-host disease. J Clin Invest (2014) 124:4266–80. doi: 10.1172/JCI75935 PMC4191032 25157821

[48] Chung J Ebens CL Perkey E Radojcic V Koch U Scarpellino L. Fibroblastic niches prime T cell alloimmunity through delta-like notch ligands. J Clin Invest (2017) 127:1574–88. doi: 10.1172/JCI89535 PMC5373885 28319044

[49] Zhang Y Sandy AR Wang J Radojcic V Shan GT Tran IT. Notch signaling is a critical regulator of allogeneic CD4+ T-cell responses mediating graft-versus-host disease. Blood (2011) 117:299–308. doi: 10.1182/blood-2010-03-271940 20870902 PMC3037751

[50] Sarantopoulos S Stevenson KE Kim HT Cutler CS Bhuiya NS Schowalter M. Altered b-cell homeostasis and excess BAFF in human chronic graft-versus-host disease. Blood (2009) 113:3865–74. doi: 10.1182/blood-2008-09-177840 PMC2670799 19168788

[51] Dehn J Spellman S Hurley CK Shaw BE Barker JN Burns LJ. Selection of unrelated donors and cord blood units for hematopoietic cell transplantation: guidelines from the NMDP/CIBMTR. Blood (2019) 134:924–34. doi: 10.1182/blood.2019001212 PMC6753623 31292117

[52] Pidala J Lee SJ Ahn KW Spellman S Wang HL Aljurf M. Nonpermissive HLA-DPB1 mismatch increases mortality after myeloablative unrelated allogeneic hematopoietic cell transplantation. Blood (2014) 124:2596–606. doi: 10.1182/blood-2014-05-576041 PMC4199961 25161269

[53] Tsamadou C Engelhardt D Platzbecker U Sala E Valerius T Wagner-Drouet E. HLA-DRB3/4/5 matching improves outcome of unrelated hematopoietic stem cell transplantation. Front Immunol (2021) 12:771449. doi: 10.3389/fimmu.2021.771449 34970261 PMC8712639

[54] Petersdorf EW Stevenson P Bengtsson M De Santis D Dubois V Gooley T. HLA-b leader and survivorship after HLA-mismatched unrelated donor transplantation. Blood (2020) 136:362–9. doi: 10.1182/blood.2020005743 PMC7365916 32483623

[55] Baron F Ruggeri A Beohou E Labopin M Mohty M Sanz J. Occurrence of graft-versus-host disease increases mortality after umbilical cord blood transplantation for acute myeloid leukaemia: A report from eurocord and the ALWP of the EBMT. J Intern Med (2018) 283:178–89. doi: 10.1111/joim.12696 28977716

[56] Feng X Kajigaya S Solomou EE Keyvanfar K Xu X Raghavachari N. Rabbit ATG but not horse ATG promotes expansion of functional CD4+CD25highFOXP3+ regulatory T cells in vitro. Blood (2008) 111:3675–83. doi: 10.1182/blood-2008-01-130146 PMC2275026 18250226

[57] Shimony O Nagler A Gellman YN Refaeli E Rosenblum N Eshkar-Sebban L. Anti-T lymphocyte globulin (ATG) induces generation of regulatory T cells, at least part of them express activated CD44. J Clin Immunol (2012) 32:173–88. doi: 10.1007/s10875-011-9599-2 21979414

[58] Champlin RE Perez WS Passweg JR Klein JP Camitta BM Gluckman E. Bone marrow transplantation for severe aplastic anemia: A randomized controlled study of conditioning regimens. Blood (2007) 109:4582–5. doi: 10.1182/blood-2006-10-052308 PMC1885491 17272503

[59] Yuan J Pei R Su W Cao J Lu Y. Meta-analysis of the actions of antithymocyte globulin in patients undergoing allogeneic hematopoietic cell transplantation. Oncotarget (2017) 8:10871–82. doi: 10.18632/oncotarget.14719 PMC5355230 28107198

[60] Naeije L Kariminia A Abdossamadi S Azadpour S Subrt P Kuzeljevic B. Anti-thymocyte globulin prophylaxis induces a decrease in naive Th cells to inhibit the onset of chronic graft-versus-Host disease: Results from the Canadian bone marrow transplant group (CBMTG) 0801 study. Biol Blood Marrow Transplant (2020) 26:438–44. doi: 10.1016/j.bbmt.2019.11.015 31756535

[61] Fujimoto A Hiramoto N Yamasaki S Inamoto Y Uchida N Maeda T. Risk factors and predictive scoring system for post-transplant lymphoproliferative disorder after hematopoietic stem cell transplantation. Biol Blood Marrow Transplant (2019) 25:1441–9. doi: 10.1016/j.bbmt.2019.02.016 30794929

[62] Admiraal R Boelens JJ. Individualized conditioning regimes in cord blood transplantation: Towards improved and predictable safety and efficacy. Expert Opin Biol Ther (2016) 16:801–13. doi: 10.1517/14712598.2016.1164688 26959558

[63] Admiraal R Lindemans CA van Kesteren C Bierings MB Versluijs AB Nierkens S. Excellent T-cell reconstitution and survival depend on low ATG exposure after pediatric cord blood transplantation. Blood (2016) 128:2734–41. doi: 10.1182/blood-2016-06-721936 27702800

[64] Admiraal R van Kesteren C Jol-van der Zijde CM Lankester AC Bierings MB Egberts TC. Association between anti-thymocyte globulin exposure and CD4+ immune reconstitution in paediatric haemopoietic cell transplantation: a multicentre, retrospective pharmacodynamic cohort analysis. Lancet Haematol (2015) 2:e194–203. doi: 10.1016/S2352-3026(15)00045-9 26688094

[65] Admiraal R van Kesteren C Jol-van der Zijde CM van Tol MJ Bartelink IH Bredius RG. Population pharmacokinetic modeling of Thymoglobulin((R)) in children receiving allogeneic-hematopoietic cell transplantation: towards improved survival through individualized dosing. Clin Pharmacokinet (2015) 54:435–46. doi: 10.1007/s40262-014-0214-6 25466602

[66] Shiratori S Ohigashi H Ara T Yasumoto A Goto H Nakagawa M. High lymphocyte counts before antithymocyte globulin administration predict acute graft-versus-host disease. Ann Hematol (2021) 100:1321–8. doi: 10.1007/s00277-020-04347-1 33215225

[67] Baron F Labopin M Blaise D Lopez-Corral L Vigouroux S Craddock C. Impact of *in vivo* T-cell depletion on outcome of AML patients in first CR given peripheral blood stem cells and reduced-intensity conditioning allo-SCT from a HLA-identical sibling donor: a report from the acute leukemia working party of the European group for blood and marrow transplantation. Bone Marrow Transplant (2014) 49:389–96. doi: 10.1038/bmt.2013.204 24419525

[68] Soiffer RJ Kim HT McGuirk J Horwitz ME Johnston L Patnaik MM. Prospective, randomized, double-blind, phase III clinical trial of anti-T-Lymphocyte globulin to assess impact on chronic graft-Versus-Host disease-free survival in patients undergoing HLA-matched unrelated myeloablative hematopoietic cell transplantation. J Clin Oncol (2017) 35:4003–11. doi: 10.1200/JCO.2017.75.8177 PMC8462523 29040031

[69] Socie G Schmoor C Bethge WA Ottinger HD Stelljes M Zander AR. Chronic graft-versus-host disease: Long-term results from a randomized trial on graft-versus-host disease prophylaxis with or without anti-t-cell globulin ATG-fresenius. Blood (2011) 117:6375–82. doi: 10.1182/blood-2011-01-329821 21467544

[70] Kroger N Solano C Wolschke C Bandini G Patriarca F Pini M. Antilymphocyte globulin for prevention of chronic graft-versus-Host disease. N Engl J Med (2016) 374:43–53. doi: 10.1056/NEJMoa1506002 26735993

[71] Chang YJ Wu DP Lai YR Liu QF Sun YQ Hu J. Antithymocyte Globulin for Matched Sibling Donor Transplantation in Patients With Hematologic Malignancies: A Multicenter, Open-Label, Randomized Controlled Study. J Clin Oncol (2020). doi: 10.1200/JCO.20.00150 32650683

[72] Walker I Panzarella T Couban S Couture F Devins G Elemary M. Pretreatment with anti-thymocyte globulin versus no anti-thymocyte globulin in patients with haematological malignancies undergoing haemopoietic cell transplantation from unrelated donors: A randomised, controlled, open-label, phase 3, multicentre trial. Lancet Oncol (2016) 17:164–73. doi: 10.1016/S1470-2045(15)00462-3 26723083

[73] Walker I Panzarella T Couban S Couture F Devins G Elemary M. Addition of anti-thymocyte globulin to standard graft-versus-host disease prophylaxis versus standard treatment alone in patients with haematological malignancies undergoing transplantation from unrelated donors: Final analysis of a randomised, open-label, multicentre, phase 3 trial. Lancet Haematol (2020) 7:e100–e11. doi: 10.1016/S2352-3026(19)30220-0 31958417

[74] Malard F Labopin M Cho C Blaise D Papadopoulos EB Passweg J. Ex vivo and *in vivo* T cell-depleted allogeneic stem cell transplantation in patients with acute myeloid leukemia in first complete remission resulted in similar overall survival: on behalf of the ALWP of the EBMT and the MSKCC. J Hematol Oncol (2018) 11:127. doi: 10.1186/s13045-018-0668-3 30342553 PMC6195954

[75] Shiratori S Sugita J Fuji S Aoki J Sawa M Ozawa Y. Low-dose antithymocyte globulin inhibits chronic graft-versus-host disease in peripheral blood stem cell transplantation from unrelated donors. Bone Marrow Transplant (2021) 56:2231–40. doi: 10.1038/s41409-021-01314-w 33963304

[76] Ali MM Gronvold B Remberger M Abrahamsen IW Myhre AE Tjonnfjord GE. Addition of anti-thymocyte globulin in allogeneic stem cell transplantation with peripheral stem cells from matched unrelated donors improves graft-Versus-Host disease and relapse free survival. Clin Lymphoma Myeloma Leuk (2021) 21:598–605. doi: 10.1016/j.clml.2021.05.003 34158268

[77] Luznik L O'Donnell PV Fuchs EJ. Post-transplantation cyclophosphamide for tolerance induction in HLA-haploidentical bone marrow transplantation. Semin Oncol (2012) 39:683–93. doi: 10.1053/j.seminoncol.2012.09.005 PMC3808078 23206845

[78] Nunes NS Kanakry CG. Mechanisms of graft-versus-Host disease prevention by post-transplantation cyclophosphamide: An evolving understanding. Front Immunol (2019) 10:2668. doi: 10.3389/fimmu.2019.02668 31849930 PMC6895959

[79] Luznik L O'Donnell PV Symons HJ Chen AR Leffell MS Zahurak M. HLA-haploidentical bone marrow transplantation for hematologic malignancies using nonmyeloablative conditioning and high-dose, posttransplantation cyclophosphamide. Biol Blood Marrow Transplant (2008) 14:641–50. doi: 10.1016/j.bbmt.2008.03.005 PMC2633246 18489989

[80] O'Donnell PV Luznik L Jones RJ Vogelsang GB Leffell MS Phelps M. Nonmyeloablative bone marrow transplantation from partially HLA-mismatched related donors using posttransplantation cyclophosphamide. Biol Blood Marrow Transplant (2002) 8:377–86. doi: 10.1053/bbmt.2002.v8.pm12171484 12171484

[81] Bolanos-Meade J Reshef R Fraser R Fei M Abhyankar S Al-Kadhimi Z. Three prophylaxis regimens (tacrolimus, mycophenolate mofetil, and cyclophosphamide; tacrolimus, methotrexate, and bortezomib; or tacrolimus, methotrexate, and maraviroc) versus tacrolimus and methotrexate for prevention of graft-versus-host disease with haemopoietic cell transplantation with reduced-intensity conditioning: A randomised phase 2 trial with a non-randomised contemporaneous control group (BMT CTN 1203). Lancet Haematol (2019) 6:e132–e43. doi: 10.1016/S2352-3026(18)30221-7 PMC6503965 30824040

[82] Brunstein CG Fuchs EJ Carter SL Karanes C Costa LJ Wu J. Alternative donor transplantation after reduced intensity conditioning: results of parallel phase 2 trials using partially HLA-mismatched related bone marrow or unrelated double umbilical cord blood grafts. Blood (2011) 118:282–8. doi: 10.1182/blood-2011-03-344853 PMC3138683 21527516

[83] Fuchs EJ O'Donnell PV Eapen M Logan B Antin JH Dawson P. Double unrelated umbilical cord blood vs HLA-haploidentical bone marrow transplantation: the BMT CTN 1101 trial. Blood (2021) 137:420–8. doi: 10.1182/blood.2020007535 PMC7819761 33475736

[84] Gooptu M Romee R St Martin A Arora M Al Malki M Antin JH. HLA-haploidentical vs matched unrelated donor transplants with posttransplant cyclophosphamide-based prophylaxis. Blood (2021) 138:273–82. doi: 10.1182/blood.2021011281 PMC8310426 34292325

[85] Munchel A Kesserwan C Symons HJ Luznik L Kasamon YL Jones RJ. Nonmyeloablative, HLA-haploidentical bone marrow transplantation with high dose, post-transplantation cyclophosphamide. Pediatr Rep (2011) 3 Suppl 2:e15. doi: 10.4081/pr.2011.s2.e15 PMC3206539 22053277

[86] Dulery R Bastos J Paviglianiti A Malard F Brissot E Battipaglia G. Thiotepa, busulfan, and fludarabine conditioning regimen in T cell-replete HLA-haploidentical hematopoietic stem cell transplantation. Biol Blood Marrow Transplant (2019) 25:1407–15. doi: 10.1016/j.bbmt.2019.02.025 30871978

[87] Hong KT Kang HJ Choi JY Hong CR Cheon JE Park JD. Favorable outcome of post-transplantation cyclophosphamide haploidentical peripheral blood stem cell transplantation with targeted busulfan-based myeloablative conditioning using intensive pharmacokinetic monitoring in pediatric patients. Biol Blood Marrow Transplant (2018) 24:2239–44. doi: 10.1016/j.bbmt.2018.06.034 29981849

[88] Solomon SR Sizemore CA Sanacore M Zhang X Brown S Holland HK. Total body irradiation-based myeloablative haploidentical stem cell transplantation is a safe and effective alternative to unrelated donor transplantation in patients without matched sibling donors. Biol Blood Marrow Transplant (2015) 21:1299–307. doi: 10.1016/j.bbmt.2015.03.003 25797174

[89] Symons HJ Zahurak M Cao Y Chen A Cooke K Gamper C. Myeloablative haploidentical BMT with posttransplant cyclophosphamide for hematologic malignancies in children and adults. Blood Adv (2020) 4:3913–25. doi: 10.1182/bloodadvances.2020001648 PMC7448587 32813874

[90] Solomon SR Sizemore CA Sanacore M Zhang X Brown S Holland HK. Haploidentical transplantation using T cell replete peripheral blood stem cells and myeloablative conditioning in patients with high-risk hematologic malignancies who lack conventional donors is well tolerated and produces excellent relapse-free survival: results of a prospective phase II trial. Biol Blood Marrow Transplant (2012) 18:1859–66. doi: 10.1016/j.bbmt.2012.06.019 22863841

[91] Mielcarek M Furlong T O'Donnell PV Storer BE McCune JS Storb R. Posttransplantation cyclophosphamide for prevention of graft-versus-host disease after HLA-matched mobilized blood cell transplantation. Blood (2016) 127:1502–8. doi: 10.1182/blood-2015-10-672071 PMC4797026 26764356

[92] McCurdy SR Kanakry CG Tsai HL Gojo I Smith BD Gladstone DE. Development of grade II acute graft-versus-Host disease is associated with improved survival after myeloablative HLA-matched bone marrow transplantation using single-agent post-transplant cyclophosphamide. Biol Blood Marrow Transplant (2019) 25:1128–35. doi: 10.1016/j.bbmt.2018.12.767 PMC6559825 30599208

[93] Kanakry CG O'Donnell PV Furlong T de Lima MJ Wei W Medeot M. Multi-institutional study of post-transplantation cyclophosphamide as single-agent graft-versus-host disease prophylaxis after allogeneic bone marrow transplantation using myeloablative busulfan and fludarabine conditioning. J Clin Oncol (2014) 32:3497–505. doi: 10.1200/JCO.2013.54.0625 PMC4209101 25267759

[94] Bashey A Zhang X Jackson K Brown S Ridgeway M Solh M. Comparison of outcomes of hematopoietic cell transplants from T-replete haploidentical donors using post-transplantation cyclophosphamide with 10 of 10 HLA-a, -b, -c, -DRB1, and -DQB1 allele-matched unrelated donors and HLA-identical sibling donors: A multivariable analysis including disease risk index. Biol Blood Marrow Transplant (2016) 22:125–33. doi: 10.1016/j.bbmt.2015.09.002 26359881

[95] Mehta RS Saliba RM Alsfeld LC Jorgensen JL Wang SA Anderlini P. Bone marrow versus peripheral blood grafts for haploidentical hematopoietic cell transplantation with post-transplantation cyclophosphamide. Transplant Cell Ther (2021) 27:1003 e1– e13. doi: 10.1016/j.jtct.2021.09.003 PMC8504778 34537419

[96] Ruggeri A Labopin M Bacigalupo A Gulbas Z Koc Y Blaise D. Bone marrow versus mobilized peripheral blood stem cells in haploidentical transplants using posttransplantation cyclophosphamide. Cancer (2018) 124:1428–37. doi: 10.1002/cncr.31228 29360162

[97] Nagler A Dholaria B Labopin M Savani BN Angelucci E Koc Y. Bone marrow versus mobilized peripheral blood stem cell graft in T-cell-replete haploidentical transplantation in acute lymphoblastic leukemia. Leukemia (2020) 34:2766–75. doi: 10.1038/s41375-020-0850-9 32393841

[98] Bashey A Zhang MJ McCurdy SR St Martin A Argall T Anasetti C. Mobilized peripheral blood stem cells versus unstimulated bone marrow as a graft source for T-Cell-Replete haploidentical donor transplantation using post-transplant cyclophosphamide. J Clin Oncol (2017) 35:3002–9. doi: 10.1200/JCO.2017.72.8428 PMC5590802 28644773

[99] Goldsmith SR Abid MB Auletta JJ Bashey A Beitinjaneh A Castillo P. Posttransplant cyclophosphamide is associated with increased cytomegalovirus infection: A CIBMTR analysis. Blood (2021) 137:3291–305. doi: 10.1182/blood.2020009362 PMC8351903 33657221

[100] Raiola AM Dominietto A di Grazia C Lamparelli T Gualandi F Ibatici A. Unmanipulated haploidentical transplants compared with other alternative donors and matched sibling grafts. Biol Blood Marrow Transplant (2014) 20:1573–9. doi: 10.1016/j.bbmt.2014.05.029 24910379

[101] Singh A Dandoy CE Chen M Kim S Mulroney CM Kharfan-Dabaja MA. Post-transplantation cyclophosphamide is associated with an increase in non-cytomegalovirus herpesvirus infections in patients with acute leukemia and myelodysplastic syndrome. Transplant Cell Ther (2022) 28:48 e1– e10. doi: 10.1016/j.jtct.2021.09.015 PMC9717499 34587551

[102] Bolanos-Meade J Cooke KR Gamper CJ Ali SA Ambinder RF Borrello IM. Effect of increased dose of total body irradiation on graft failure associated with HLA-haploidentical transplantation in patients with severe haemoglobinopathies: a prospective clinical trial. Lancet Haematol (2019) 6:e183–e93. doi: 10.1016/S2352-3026(19)30031-6 PMC6506220 30878319

[103] Bolanos-Meade J Fuchs EJ Luznik L Lanzkron SM Gamper CJ Jones RJ. HLA-haploidentical bone marrow transplantation with posttransplant cyclophosphamide expands the donor pool for patients with sickle cell disease. Blood (2012) 120:4285–91. doi: 10.1182/blood-2012-07-438408 PMC3507140 22955919

[104] Li Y Wang N Li L Cao Y Xu J Wang J. Haploidentical transplantation with modified post-transplantation cyclophosphamide for patients with primary aplastic anemia: A multicenter experience. Transplant Cell Ther (2021) 27:331 e1– e7. doi: 10.1016/j.jtct.2021.01.018 33836879

[105] Fernandes JF Nichele S Arcuri LJ Ribeiro L Zamperlini-Netto G Loth G. Outcomes after haploidentical stem cell transplantation with post-transplantation cyclophosphamide in patients with primary immunodeficiency diseases. Biol Blood Marrow Transplant (2020) 26:1923–9. doi: 10.1016/j.bbmt.2020.07.003 32653621

[106] DeZern AE Zahurak ML Symons HJ Cooke KR Rosner GL Gladstone DE. Haploidentical BMT for severe aplastic anemia with intensive GVHD prophylaxis including posttransplant cyclophosphamide. Blood Adv (2020) 4:1770–9. doi: 10.1182/bloodadvances.2020001729 PMC7189283 32343796

[107] Bonfim C Ribeiro L Nichele S Loth G Bitencourt M Koliski A. Haploidentical bone marrow transplantation with post-transplant cyclophosphamide for children and adolescents with fanconi anemia. Biol Blood Marrow Transplant (2017) 23:310–7. doi: 10.1016/j.bbmt.2016.11.006 27832981

[108] Arcuri LJ Nabhan SK Cunha R Nichele S Ribeiro AAF Fernandes JF. Impact of CD34 cell dose and conditioning regimen on outcomes after haploidentical donor hematopoietic stem cell transplantation with post-transplantation cyclophosphamide for Relapsed/Refractory severe aplastic anemia. Biol Blood Marrow Transplant (2020) 26:2311–7. doi: 10.1016/j.bbmt.2020.09.007 32949751

[109] Dulery R Mohty R Labopin M Sestili S Malard F Brissot E. Early cardiac toxicity associated with post-transplant cyclophosphamide in allogeneic stem cell transplantation. JACC CardioOncol (2021) 3:250–9. doi: 10.1016/j.jaccao.2021.02.011 PMC8352028 34396331

[110] Yeh ET Tong AT Lenihan DJ Yusuf SW Swafford J Champion C. Cardiovascular complications of cancer therapy: diagnosis, pathogenesis, and management. Circulation (2004) 109:3122–31. doi: 10.1161/01.CIR.0000133187.74800.B9 15226229

[111] Moreland L Bate G Kirkpatrick P. Abatacept. Nat Rev Drug Discovery (2006) 5:185–6. doi: 10.1038/nrd1989 16557658

[112] Koura DT Horan JT Langston AA Qayed M Mehta A Khoury HJ. *In vivo* T cell costimulation blockade with abatacept for acute graft-versus-host disease prevention: a first-in-disease trial. Biol Blood Marrow Transplant (2013) 19:1638–49. doi: 10.1016/j.bbmt.2013.09.003 24047754

[113] Watkins B Qayed M McCracken C Bratrude B Betz K Suessmuth Y. Phase II trial of costimulation blockade with abatacept for prevention of acute GVHD. J Clin Oncol (2021) 39:1865–77. doi: 10.1200/JCO.20.01086 PMC8260909 33449816

[114] Ngwube A Shah N Godder K Jacobsohn D Hulbert ML Shenoy S. Abatacept is effective as GVHD prophylaxis in unrelated donor stem cell transplantation for children with severe sickle cell disease. Blood Adv (2020) 4:3894–9. doi: 10.1182/bloodadvances.2020002236 PMC7448594 32813873

[115] Devine SM Carter S Soiffer RJ Pasquini MC Hari PN Stein A. Low risk of chronic graft-versus-host disease and relapse associated with T cell-depleted peripheral blood stem cell transplantation for acute myelogenous leukemia in first remission: results of the blood and marrow transplant clinical trials network protocol 0303. Biol Blood Marrow Transplant (2011) 17:1343–51. doi: 10.1016/j.bbmt.2011.02.002 PMC3150599 21320619

[116] Barba P Hilden P Devlin SM Maloy M Dierov D Nieves J. Ex vivo CD34(+)-selected T cell-depleted peripheral blood stem cell grafts for allogeneic hematopoietic stem cell transplantation in acute leukemia and myelodysplastic syndrome is associated with low incidence of acute and chronic graft-versus-Host disease and high treatment response. Biol Blood Marrow Transplant (2017) 23:452–8. doi: 10.1016/j.bbmt.2016.12.633 PMC5398850 28017734

[117] Pasquini MC Devine S Mendizabal A Baden LR Wingard JR Lazarus HM. Comparative outcomes of donor graft CD34+ selection and immune suppressive therapy as graft-versus-host disease prophylaxis for patients with acute myeloid leukemia in complete remission undergoing HLA-matched sibling allogeneic hematopoietic cell transplantation. J Clin Oncol (2012) 30:3194–201. doi: 10.1200/JCO.2012.41.7071 PMC3434978 22869882

[118] Peters C Matthes-Martin S Fritsch G Holter W Lion T Witt V. Transplantation of highly purified peripheral blood CD34+ cells from HLA-mismatched parental donors in 14 children: evaluation of early monitoring of engraftment. Leukemia (1999) 13:2070–8. doi: 10.1038/sj.leu.2401577 PMC7101968 10602431

[119] Zecca M Strocchio L Pagliara D Comoli P Bertaina A Giorgiani G. HLA-haploidentical T cell-depleted allogeneic hematopoietic stem cell transplantation in children with fanconi anemia. Biol Blood Marrow Transplant (2014) 20:571–6. doi: 10.1016/j.bbmt.2014.01.015 24462983

[120] Chen BJ Cui X Sempowski GD Liu C Chao NJ. Transfer of allogeneic CD62L- memory T cells without graft-versus-host disease. Blood (2004) 103:1534–41. doi: 10.1182/blood-2003-08-2987 14551132

[121] Chen BJ Deoliveira D Cui X Le NT Son J Whitesides JF. Inability of memory T cells to induce graft-versus-host disease is a result of an abortive alloresponse. Blood (2007) 109:3115–23. doi: 10.1182/blood-2006-04-016410 PMC1852216 17148592

[122] Zhang J Barefoot BE Mo W Deoliveira D Son J Cui X. CD62L- memory T cells enhance T-cell regeneration after allogeneic stem cell transplantation by eliminating host resistance in mice. Blood (2012) 119:6344–53. doi: 10.1182/blood-2011-03-342055 PMC3383193 22596261

[123] Bleakley M Heimfeld S Jones LA Turtle C Krause D Riddell SR. Engineering human peripheral blood stem cell grafts that are depleted of naive T cells and retain functional pathogen-specific memory T cells. Biol Blood Marrow Transplant (2014) 20:705–16. doi: 10.1016/j.bbmt.2014.01.032 PMC3985542 24525279

[124] Bleakley M Otterud BE Richardt JL Mollerup AD Hudecek M Nishida T. Leukemia-associated minor histocompatibility antigen discovery using T-cell clones isolated by *in vitro* stimulation of naive CD8+ T cells. Blood (2010) 115:4923–33. doi: 10.1182/blood-2009-12-260539 PMC2890170 20203263

[125] Bleakley M Sehgal A Seropian S Biernacki MA Krakow EF Dahlberg A. Naive T-cell depletion to prevent chronic graft-Versus-Host disease. J Clin Oncol (2022) 40:1174–85. doi: 10.1200/JCO.21.01755 PMC8987226 35007144

[126] Bleakley M Heimfeld S Loeb KR Jones LA Chaney C Seropian S. Outcomes of acute leukemia patients transplanted with naive T cell-depleted stem cell grafts. J Clin Invest (2015) 125:2677–89. doi: 10.1172/JCI81229 PMC4563691 26053664

[127] Triplett BM Shook DR Eldridge P Li Y Kang G Dallas M. Rapid memory T-cell reconstitution recapitulating CD45RA-depleted haploidentical transplant graft content in patients with hematologic malignancies. Bone Marrow Transplant (2015) 50:1012. doi: 10.1038/bmt.2015.139 26130176

[128] Sisinni L Gasior M de Paz R Querol S Bueno D Fernandez L. Unexpected high incidence of human herpesvirus-6 encephalitis after naive T cell-depleted graft of haploidentical stem cell transplantation in pediatric patients. Biol Blood Marrow Transplant (2018) 24:2316–23. doi: 10.1016/j.bbmt.2018.07.016 30031939

[129] Merli P Algeri M Galaverna F Milano GM Bertaina V Biagini S. Immune modulation properties of zoledronic acid on TcRgammadelta T-lymphocytes after TcRalphabeta/CD19-depleted haploidentical stem cell transplantation: An analysis on 46 pediatric patients affected by acute leukemia. Front Immunol (2020) 11:699. doi: 10.3389/fimmu.2020.00699 32477328 PMC7235359

[130] Tsilifis C Lum SH Nademi Z Hambleton S Flood TJ Williams EJ. TCRalphabeta-depleted haploidentical grafts are a safe alternative to HLA-matched unrelated donor stem cell transplants for infants with severe combined immunodeficiency. J Clin Immunol (2022) 42:851–8. doi: 10.1007/s10875-022-01239-z PMC9166847 35305204

[131] Lum SH Greener S Perez-Heras I Drozdov D Payne RP Watson H. T-Replete HLA-matched grafts vs T-depleted HLA-mismatched grafts in inborn errors of immunity. Blood Adv (2022) 6:1319–28. doi: 10.1182/bloodadvances.2020004072 PMC8864655 34972212

[132] Shah RM Elfeky R Nademi Z Qasim W Amrolia P Chiesa R. T-Cell receptor alphabeta(+) and CD19(+) cell-depleted haploidentical and mismatched hematopoietic stem cell transplantation in primary immune deficiency. J Allergy Clin Immunol (2018) 141:1417–26 e1. doi: 10.1016/j.jaci.2017.07.008 28780238

[133] Strocchio L Pagliara D Algeri M Li Pira G Rossi F Bertaina V. HLA-haploidentical TCRalphabeta+/CD19+-depleted stem cell transplantation in children and young adults with fanconi anemia. Blood Adv (2021) 5:1333–9. doi: 10.1182/bloodadvances.2020003707 PMC7948273 33656536

[134] Gaziev J Isgro A Sodani P Paciaroni K De Angelis G Marziali M. Haploidentical HSCT for hemoglobinopathies: Improved outcomes with TCRalphabeta(+)/CD19(+)-depleted grafts. Blood Adv (2018) 2:263–70. doi: 10.1182/bloodadvances.2017012005 PMC5812329 29431621

[135] Maschan M Shelikhova L Ilushina M Shekhovtsova Z Khismatullina R Kurnikova E. Outcome of alphabeta T cell-depleted transplantation in children with high-risk acute myeloid leukemia, grafted in remission. Bone Marrow Transplant (2020) 55:256–9. doi: 10.1038/s41409-019-0531-3 30988381

[136] Leahy AB Li Y Talano JA Elgarten CW Seif AE Wang Y. Unrelated donor alpha/beta T cell- and b cell-depleted HSCT for the treatment of pediatric acute leukemia. Blood Adv (2022) 6:1175–85. doi: 10.1182/bloodadvances.2021005492 PMC8864664 34872106

[137] Bertaina A Zecca M Buldini B Sacchi N Algeri M Saglio F. Unrelated donor vs HLA-haploidentical alpha/beta T-cell- and b-cell-depleted HSCT in children with acute leukemia. Blood (2018) 132:2594–607. doi: 10.1182/blood-2018-07-861575 30348653

[138] Merli P Pagliara D Galaverna F Li Pira G Andreani M Leone G. TCRalphabeta/CD19 depleted HSCT from an HLA-haploidentical relative to treat children with different nonmalignant disorders. Blood Adv (2022) 6:281–92. doi: 10.1182/bloodadvances.2021005628 PMC8753220 34592755

[139] Laberko A Sultanova E Gutovskaya E Shipitsina I Shelikhova L Kurnikova E. Mismatched related vs matched unrelated donors in TCRalphabeta/CD19-depleted HSCT for primary immunodeficiencies. Blood (2019) 134:1755–63. doi: 10.1182/blood.2019001757 PMC6856988 31558465

[140] de Witte MA Janssen A Nijssen K Karaiskaki F Swanenberg L van Rhenen A. Alphabeta T-cell graft depletion for allogeneic HSCT in adults with hematological malignancies. Blood Adv (2021) 5:240–9. doi: 10.1182/bloodadvances.2020002444 PMC7805311 33570642

[141] Locatelli F Merli P Pagliara D Li Pira G Falco M Pende D. Outcome of children with acute leukemia given HLA-haploidentical HSCT after alphabeta T-cell and b-cell depletion. Blood (2017) 130:677–85. doi: 10.1182/blood-2017-04-779769 28588018

[142] Giardino S Bagnasco F Falco M Miano M Pierri F Risso M. Haploidentical stem cell transplantation after TCR-alphabeta(+) and CD19(+) cells depletion in children with congenital non-malignant disease. Transplant Cell Ther (2022) 28:394 e1–e9. doi: 10.1016/j.jtct.2022.04.002 35405368

[143] Yeshurun M Weisdorf D Rowe JM Tallman MS Zhang MJ Wang HL. The impact of the graft-versus-leukemia effect on survival in acute lymphoblastic leukemia. Blood Adv (2019) 3:670–80. doi: 10.1182/bloodadvances.2018027003 PMC6391668 30808685

[144] Bona K Brazauskas R He N Lehmann L Abdel-Azim H Ahmed IA. Neighborhood poverty and pediatric allogeneic hematopoietic cell transplantation outcomes: A CIBMTR analysis. Blood (2021) 137:556–68. doi: 10.1182/blood.2020006252 PMC7845011 33104215

[145] Shelikhova L Ilushina M Shekhovtsova Z Shasheleva D Khismatullina R Kurnikova E. Alphabeta T cell-depleted haploidentical hematopoietic stem cell transplantation without antithymocyte globulin in children with chemorefractory acute myelogenous leukemia. Biol Blood Marrow Transplant (2019) 25:e179–e82. doi: 10.1016/j.bbmt.2019.01.023 30677509

[146] Bertaina A Zorzoli A Petretto A Barbarito G Inglese E Merli P. Zoledronic acid boosts gammadelta T-cell activity in children receiving alphabeta(+) T and CD19(+) cell-depleted grafts from an HLA-haplo-identical donor. Oncoimmunology (2017) 6:e1216291. doi: 10.1080/2162402X.2016.1216291 28344861 PMC5353934

[147] Laberko A Bogoyavlenskaya A Shelikhova L Shekhovtsova Z Balashov D Voronin K. Risk factors for and the clinical impact of cytomegalovirus and Epstein-Barr virus infections in pediatric recipients of TCR-alpha/beta- and CD19-depleted grafts. Biol Blood Marrow Transplant (2017) 23:483–90. doi: 10.1016/j.bbmt.2016.12.635 28039080

[148] Gluckman E Broxmeyer HA Auerbach AD Friedman HS Douglas GW Devergie A. Hematopoietic reconstitution in a patient with fanconi's anemia by means of umbilical-cord blood from an HLA-identical sibling. N Engl J Med (1989) 321:1174–8. doi: 10.1056/NEJM198910263211707 2571931

[149] Zhu X Tang B Sun Z. Umbilical cord blood transplantation: Still growing and improving. Stem Cells Transl Med (2021) 10 Suppl 2:S62–74. doi: 10.1002/sctm.20-0495 PMC8560197 34724722

[150] Brown JA Boussiotis VA. Umbilical cord blood transplantation: basic biology and clinical challenges to immune reconstitution. Clin Immunol (2008) 127:286–97. doi: 10.1016/j.clim.2008.02.008 PMC2468219 18395491

[151] da Silva CL Goncalves R Porada CD Ascensao JL Zanjani ED Cabral JM. Differences amid bone marrow and cord blood hematopoietic stem/progenitor cell division kinetics. J Cell Physiol (2009) 220:102–11. doi: 10.1002/jcp.21736 PMC4008884 19277981

[152] Theunissen K Verfaillie CM. A multifactorial analysis of umbilical cord blood, adult bone marrow and mobilized peripheral blood progenitors using the improved ML-IC assay. Exp Hematol (2005) 33:165–72. doi: 10.1016/j.exphem.2004.10.016 15676210

[153] Kurtzberg J Laughlin M Graham ML Smith C Olson JF Halperin EC. Placental blood as a source of hematopoietic stem cells for transplantation into unrelated recipients. N Engl J Med (1996) 335:157–66. doi: 10.1056/NEJM199607183350303 8657213

[154] Eapen M Rubinstein P Zhang MJ Stevens C Kurtzberg J Scaradavou A. Outcomes of transplantation of unrelated donor umbilical cord blood and bone marrow in children with acute leukaemia: A comparison study. Lancet (2007) 369:1947–54. doi: 10.1016/S0140-6736(07)60915-5 17560447

[155] Rocha V Cornish J Sievers EL Filipovich A Locatelli F Peters C. Comparison of outcomes of unrelated bone marrow and umbilical cord blood transplants in children with acute leukemia. Blood (2001) 97:2962–71. doi: 10.1182/blood.V97.10.2962 11342418

[156] Barker JN Doubrovina E Sauter C Jaroscak JJ Perales MA Doubrovin M. Successful treatment of EBV-associated posttransplantation lymphoma after cord blood transplantation using third-party EBV-specific cytotoxic T lymphocytes. Blood (2010) 116:5045–9. doi: 10.1182/blood-2010-04-281873 PMC3012598 20826724

[157] Eapen M Klein JP Sanz GF Spellman S Ruggeri A Anasetti C. Effect of donor-recipient HLA matching at HLA a, b, c, and DRB1 on outcomes after umbilical-cord blood transplantation for leukaemia and myelodysplastic syndrome: a retrospective analysis. Lancet Oncol (2011) 12:1214–21. doi: 10.1016/S1470-2045(11)70260-1 PMC3245836 21982422

[158] Gluckman E Rocha V Arcese W Michel G Sanz G Chan KW. Factors associated with outcomes of unrelated cord blood transplant: Guidelines for donor choice. Exp Hematol (2004) 32:397–407. doi: 10.1016/j.exphem.2004.01.002 15050751

[159] Ruggeri A Eapen M Scaravadou A Cairo MS Bhatia M Kurtzberg J. Umbilical cord blood transplantation for children with thalassemia and sickle cell disease. Biol Blood Marrow Transplant (2011) 17:1375–82. doi: 10.1016/j.bbmt.2011.01.012 PMC3395002 21277376

[160] Gluckman E Rocha V Ionescu I Bierings M Harris RE Wagner J. Results of unrelated cord blood transplant in fanconi anemia patients: risk factor analysis for engraftment and survival. Biol Blood Marrow Transplant (2007) 13:1073–82. doi: 10.1016/j.bbmt.2007.05.015 17697970

[161] Page KM Labopin M Ruggeri A Michel G Diaz de Heredia C O'Brien T. Factors associated with long-term risk of relapse after unrelated cord blood transplantation in children with acute lymphoblastic leukemia in remission. Biol Blood Marrow Transplant (2017) 23:1350–8. doi: 10.1016/j.bbmt.2017.04.015 PMC5569913 28438676

[162] Robin M Ruggeri A Labopin M Niederwieser D Tabrizi R Sanz G. Comparison of unrelated cord blood and peripheral blood stem cell transplantation in adults with myelodysplastic syndrome after reduced-intensity conditioning regimen: a collaborative study from eurocord (Cord blood committee of cellular therapy & immunobiology working party of EBMT) and chronic malignancies working party. Biol Blood Marrow Transplant (2015) 21:489–95. doi: 10.1016/j.bbmt.2014.11.675 25529382

[163] Gerds AT Woo Ahn K Hu ZH Abdel-Azim H Akpek G Aljurf M. Outcomes after umbilical cord blood transplantation for myelodysplastic syndromes. Biol Blood Marrow Transplant (2017) 23:971–9. doi: 10.1016/j.bbmt.2017.03.014 PMC5474679 28288952

[164] Tong J Xuan L Sun Y Huang D Liu H Zheng C. Umbilical cord blood transplantation without antithymocyte globulin results in similar survival but better quality of life compared with unrelated peripheral blood stem cell transplantation for the treatment of acute leukemia-a retrospective study in China. Biol Blood Marrow Transplant (2017) 23:1541–8. doi: 10.1016/j.bbmt.2017.05.004 28499936

[165] Zheng CC Zhu XY Tang BL Zhang XH Zhang L Geng LQ. Clinical separation of cGvHD and GvL and better GvHD-free/relapse-free survival (GRFS) after unrelated cord blood transplantation for AML. Bone Marrow Transplant (2017) 52:88–94. doi: 10.1038/bmt.2016.182 27376453

[166] Sharma P Purev E Haverkos B Pollyea DA Cherry E Kamdar M. Adult cord blood transplant results in comparable overall survival and improved GRFS vs matched related transplant. Blood Adv (2020) 4:2227–35. doi: 10.1182/bloodadvances.2020001554 PMC7252552 32442301

[167] Balligand L Galambrun C Sirvent A Roux C Pochon C Bruno B. Single-unit versus double-unit umbilical cord blood transplantation in children and young adults with residual leukemic disease. Biol Blood Marrow Transplant (2019) 25:734–42. doi: 10.1016/j.bbmt.2018.10.016 30385256

[168] Kindwall-Keller TL Hegerfeldt Y Meyerson HJ Margevicius S Fu P van Heeckeren W. Prospective study of one- vs two-unit umbilical cord blood transplantation following reduced intensity conditioning in adults with hematological malignancies. Bone Marrow Transplant (2012) 47:924–33. doi: 10.1038/bmt.2011.195 PMC3262108 22002488

[169] Michel G Galambrun C Sirvent A Pochon C Bruno B Jubert C. Single- vs double-unit cord blood transplantation for children and young adults with acute leukemia or myelodysplastic syndrome. Blood (2016) 127:3450–7. doi: 10.1182/blood-2016-01-694349 27099151

[170] Wagner JE Jr. Eapen M Carter S Wang Y Schultz KR Wall DA. One-unit versus two-unit cord-blood transplantation for hematologic cancers. N Engl J Med (2014) 371:1685–94. doi: 10.1056/NEJMoa1405584 PMC4257059 25354103

[171] Hiwarkar P Qasim W Ricciardelli I Gilmour K Quezada S Saudemont A. Cord blood T cells mediate enhanced antitumor effects compared with adult peripheral blood T cells. Blood (2015) 126:2882–91. doi: 10.1182/blood-2015-06-654780 26450984

[172] Milano F Appelbaum FR Delaney C. Cord-blood transplantation in patients with minimal residual disease. N Engl J Med (2016) 375:2204–5. doi: 10.1056/NEJMoa1602074 27959748

[173] Servais S Hannon M Peffault de Latour R Socie G Beguin Y. Reconstitution of adaptive immunity after umbilical cord blood transplantation: impact on infectious complications. Stem Cell Investig (2017) 4:40. doi: 10.21037/sci.2017.05.03 PMC5460101 28607914

[174] Karantanos T Kim HT Tijaro-Ovalle NM Li L Cutler C Antin JH. Reactivation of BK virus after double umbilical cord blood transplantation in adults correlates with impaired reconstitution of CD4(+) and CD8(+) T effector memory cells and increase of T regulatory cells. Clin Immunol (2019) 207:18–23. doi: 10.1016/j.clim.2019.06.010 31255803 PMC8091796

[175] Linder KA McDonald PJ Kauffman CA Revankar SG Chandrasekar PH Miceli MH. Infectious complications after umbilical cord blood transplantation for hematological malignancy. Open Forum Infect Dis (2019) 6:ofz037. doi: 10.1093/ofid/ofz037 30815505 PMC6386816

[176] Saavedra S Sanz GF Jarque I Moscardo F Jimenez C Lorenzo I. Early infections in adult patients undergoing unrelated donor cord blood transplantation. Bone Marrow Transplant (2002) 30:937–43. doi: 10.1038/sj.bmt.1703764 12476288

[177] Lindemans CA Chiesa R Amrolia PJ Rao K Nikolajeva O de Wildt A. Impact of thymoglobulin prior to pediatric unrelated umbilical cord blood transplantation on immune reconstitution and clinical outcome. Blood (2014) 123:126–32. doi: 10.1182/blood-2013-05-502385 24184682

[178] Pascal L Tucunduva L Ruggeri A Blaise D Ceballos P Chevallier P. Impact of ATG-containing reduced-intensity conditioning after single- or double-unit allogeneic cord blood transplantation. Blood (2015) 126:1027–32. doi: 10.1182/blood-2014-09-599241 26160301

[179] Horowitz MM Gale RP Sondel PM Goldman JM Kersey J Kolb HJ. Graft-versus-leukemia reactions after bone marrow transplantation. Blood (1990) 75:555–62. doi: 10.1182/blood.V75.3.555.555 2297567

